# Platelet cloaking of circulating tumour cells in patients with metastatic prostate cancer: Results from ExPeCT, a randomised controlled trial

**DOI:** 10.1371/journal.pone.0243928

**Published:** 2020-12-18

**Authors:** Lauren Brady, Brian Hayes, Gráinne Sheill, Anne-Marie Baird, Emer Guinan, Bryan Stanfill, Tatjana Vlajnic, Orla Casey, Verena Murphy, John Greene, Emma H. Allott, Juliette Hussey, Fidelma Cahill, Mieke Van Hemelrijck, Nicola Peat, Lorelei Mucci, Moya Cunningham, Liam Grogan, Thomas Lynch, Rustom P. Manecksha, John McCaffrey, Dearbhaile O’Donnell, Orla Sheils, John O’Leary, Sarah Rudman, Ray McDermott, Stephen Finn

**Affiliations:** 1 Department of Histopathology and Morbid Anatomy, Trinity Translational Medicine Institute, Trinity College Dublin, Dublin, Ireland; 2 Department of Histopathology, Cork University Hospital, Cork, Ireland; 3 Department of Pathology, University College Cork, Cork, Ireland; 4 Discipline of Physiotherapy, School of Medicine, Trinity College Dublin, Dublin, Ireland; 5 School of Medicine, Trinity College Dublin, Dublin, Ireland; 6 Pacific Northwest National Laboratory, Richland, Washington, United States of America; 7 Institute of Pathology, University Hospital Basel, Basel, Switzerland; 8 Cancer Trials Ireland, Dublin, Ireland; 9 Centre for Cancer Research and Cell Biology, Queen’s University Belfast, Belfast, Northern Ireland, United Kingdom; 10 King’s College London, School of Cancer and Pharmaceutical Sciences, Translational Oncology and Urology (TOUR), London, United Kingdom; 11 Guy’s and St Thomas’ NHS Foundation Trust, London, United Kingdom; 12 Harvard T.H. Chan school of Public Health, Boston, Massachusetts, United States of America; 13 Department of Radiation Oncology, St Luke’s Hospital, Dublin, Ireland; 14 Department of Oncology, Beaumont Hospital, Dublin, Ireland; 15 Department of Urology, St James’s Hospital, Dublin, Ireland; 16 Department of Surgery, Trinity College Dublin, Dublin, Ireland; 17 Department of Oncology, Mater Misericordiae Hospital, Dublin, Ireland; 18 HOPE Directorate, St James’s Hospital, Dublin, Ireland; 19 Department of Histopathology, St James’s Hospital, Dublin, Ireland; 20 Department of Oncology, Tallaght University Hospital, Dublin, Ireland; Hospital de Santa Maria, PORTUGAL

## Abstract

**Background:**

Circulating tumour cells (CTCs) represent a morphologically distinct subset of cancer cells, which aid the metastatic spread. The ExPeCT trial aimed to examine the effectiveness of a structured exercise programme in modulating levels of CTCs and platelet cloaking in patients with metastatic prostate cancer.

**Methods:**

Participants (n = 61) were randomised into either standard care (control) or exercise arms. Whole blood was collected for all participants at baseline (T0), three months (T3) and six months (T6), and analysed for the presence of CTCs, CTC clusters and platelet cloaking. CTC data was correlated with clinico-pathological information.

**Results:**

Changes in CTC number were observed within group over time, however no significant difference in CTC number was observed between groups over time. Platelet cloaking was identified in 29.5% of participants. A positive correlation between CTC number and white cell count (WCC) was observed (p = 0.0001), in addition to a positive relationship between CTC clusters and PSA levels (p = 0.0393).

**Conclusion:**

The presence of platelet cloaking has been observed in this patient population for the first time, in addition to a significant correlation between CTC number and WCC.

**Trial registration:**

ClincalTrials.gov identifier NCT02453139.

## Introduction

Five-year survival rates for primary localized prostate cancer (PrCa) are high [1], however the onset of metastatic disease confers a worse prognosis resulting in a reduction in overall survival (OS) [2]. Thus, research into the development of metastatic PrCa is crucial to improve the lives of patients living with this disease.

Circulating tumour cells (CTCs) are a morphologically distinct subset of cancer cells, which possess the ability to enter the bloodstream and extravasate to distant sites, forming metastatic lesions [3]. A majority of CTCs are detected as single cells; however, CTC clusters which are cohesive aggregates of CTCs are considered to have increased invasive potential [4]. CTCs have potential use as prognostic and diagnostic liquid biopsy-based markers. A recent phase III study for patients with metastatic castrate resistant PrCa, SWOG 0421 [5], showed high levels of CTCs at baseline were associated with an elevated PSA and increased bone pain, with an increase in CTC number highly correlated with worse OS [5]. An additional study showed a 30% decrease in CTC number was associated with improved OS post treatment, suggesting CTC number as a potential surrogate marker for monitoring treatment response [6].

The mechanisms, whereby CTCs circulate undetected by the immune system, are poorly understood. One proposed mechanism relates to the ‘platelet cloaking’ of cancer cells [7]. An *in vitro* study using a combination of an ovarian cancer cell line (SK-OV-3) and platelets, demonstrated significant platelet adhesion and activation, as measured by P-selectin surface expression, and demonstrated increased invasive potential in SK-OV-3 cloaked cells [8]. Platelets can impair natural killer (NK) cell mediated cell killing [9]. In platelet depleted murine models, NK cells were effective against uncloaked NK sensitive tumour cells, resulting in a decrease in the level of spontaneous metastasis [9], thus inferring that platelet cloaking of CTCs provide a barrier to the body’s immune response.

Evidence exists, which highlights the benefits of physical activity in metastatic PrCa, however little is known about the impact of physical activity on CTCs and platelet cloaking. One study examining a cohort of men with PrCa from the Harvard Health Professionals Follow Up Study, found that moderate physical activity was attributed to prolonged OS, and vigorous activity was associated with PrCa specific survival [10]. Physical activity can reduce systemic inflammatory mediators which in turn reduce platelet activation, and an increase in NK cell number post-exercise has been observed [7]. These data give rise to the hypothesis that increased circulating NK cell numbers associated with exercise may overcome the CTC-survival enhancing effect of platelet cloaking, however more investigation is required in this area.

Based on this current evidence, the ExPeCT (Exercise, Prostate Cancer and Circulating Tumour Cells (ClincalTrials.gov identifier NCT02453139, CTrials IE 15–21)) trial was conceived to elucidate the relationship between exercise, platelet cloaking and CTCs in patients with metastatic PrCa.

## Methods

### Study population

The ExPeCT Trial protocol has previously been described elsewhere including inclusion and exclusion criteria and randomisation procedures [11]. Briefly, ExPeCT was a multi-centre (Ireland and London) prospective study designed to examine the relationship between exercise, platelet cloaking and CTCs in patients with metastatic PrCa [11]. Participants were randomised into exercise or control arms and participated in the trial for six months. Ethical approval was sought and obtained from each individual site involved in the study. The study protocol and experimental procedures were approved by NRES Committee London—Camden & Islington (REC reference 14/LO/1859 10^th^ Dec 2014), The Mater Misericordia Hospital Research Ethics Committee, Dublin (REC reference: 1/378/1760 15^th^ Jan 2014), Beaumont Hospital Ethics (Medical Research) Committee, Dublin (REC Reference 15/73 25^th^ Sept 2015), SJH/AMNCH Research Ethics Committee, Dublin (REC Reference: 2014–07 List 27(17)/2014-11 List 41 (6) 5^th^ Aug 2014) and St Luke’s Radiation Oncology Network, Dublin (REC Number not assigned. Trial referred to as ICORG15-21 (sponsorship identifier) 19^th^ Sept 2016). Written informed consent was obtained from all participants and the study was conducted in accordance with the principles of the Declaration of Helsinki. All study team members completed regular Good Clinical Practice training. Further, this study adheres to CONSORT guidelines. The authors confirm that all ongoing and related trials for this intervention are registered. The trial was registered retrospectively in May 2015 due to unforeseen and unavoidable administrative delays. The assessment and follow-up period for the primary endpoint of this study took place between October 2014 and November 2017. Each participant received a participant identifier number (PIN) and the computer programme Graphpad was used to randomly assign a treatment group to each PIN [11]. Gatekeepers informed the research team of participant allocation. The study was not blinded due to the supervised exercise component; however, study pathologists were blinded to group allocation during CTC enumeration.

### Exercise programme

The exercise programme has been described in detail previously [11]. Participants randomised into the exercise group participated in moderate-to-vigorous intensity aerobic exercise comprising a weekly class for 3 months (supervised) and home-based aerobic exercise for 3 months (unsupervised). During home-based exercise, participants were required to wear heart-rate monitors to determine adherence to the exercise prescription. Participants randomised into the control arm were encouraged to continue with their regular physical activity routines.

### CTC isolation and microscopic assessment

Whole blood was isolated at baseline (T0), three months (T3) and six months (T6). CTCs were isolated from 3 mL whole blood onto filters using size-based detection platform, ScreenCell^®^ (ScreenCell, France) (maximum 12 mL blood/4 filters per patient per time point). ScreenCell^®^ filters use a microporous membrane filter and a vacuum tube to allow leucocytes to pass through the 7.5 μm filter pores, while trapping the larger and less deformable CTCs on the filter, with good recapture rates following *in vitro* spiking experiments [12]. ScreenCell^®^ technology has been utilized in multiple different cancer types for CTC identification and enumeration, including lung [13], oesophageal [14] and colorectal [15], and a recent study confirmed the presence of non-hematological cells in all samples assessed, with multiple platelet aggregates [16]. Filters were stained using May-Grunwald Giemsa (MGG) and were examined using an Olympus BX41 light microscope (Olympus-Life Science, MA, USA). The presence (and number) of CTCs (intact CTCs/bare CTC nuclei), CTC clusters, presence or absence of platelet cloaking, were recorded per filter.

### *In vitro* platelet cloaking

In order to simulate the interaction between CTCs and platelets *in vivo*, an *in vitro* prostate cell line experiment was performed. Surplus viable human platelets were donated by the Irish Blood Transfusion Service according to an authorised clinical indemnity form. Prostate cell lines DU145 (malignant) and RWPE1 (normal) were cultured and prepared in cell suspensions to a required concentration of 1x10^6^ cells in 1 mL media. One mL platelet suspension was combined with the 1x10^6^ cells/mL suspension, immediately added to healthy donor blood and allowed to incubate at room temperature for various periods of time (5–45 min). Ten -500 μL of the platelet/cell mixture was added to whole blood and the blood was immediately fixed and filtered according to the ScreenCell^®^ filtration guidelines. A 50 μL cell/platelet suspension incubated at room temperature for 45 min produced optimum cloaking results. Platelets were present throughout the filters in aggregates on the membrane as well as in aggregates surrounding tumour cells. The total number of cells in four fields of view were counted by two independent observers, along with the number of cells displaying platelet cloaking, and the results averaged. A percentage of platelet cloaking was calculated based on the number of cloaked cells per total number of cells.

### Morphological definition of CTCs, clusters and platelet cloaking

CTCs were independently identified using stringent morphological cytology criteria by an expert cyto-pathologist (B.H.) who was blinded to trial grouping. CTCs were defined as cells in the same plane of focus as filter pores whose nucleus was at least twice the diameter of a filter pore, dark blue / purple in colour and with an outline which was well-defined around its entire circumference. CTCs lacking a cytoplasm were considered “bare CTC nuclei”. A CTC cluster was defined as a cohesive group of two or more cells which individually achieved the diagnostic criteria for CTCs. Platelet cloaking of an individual CTC was defined as the presence of at least one platelet in direct contact with the edge of the CTC.

### Statistical analysis

Sample size and study power have been described previously in the study protocol [11]. Briefly, the study aimed to recruit 200 participants over the lifetime of the study, evenly divided between the exercise group and the control group. Study power was calculated based on data from a study of ovarian cancer cell lines [11, 17], which showed approximately 2% platelet adhesion and a study of PrCa CTCs [18] that determined a mean of 75 CTCs with a standard deviation (SD) of 333. If the SD of platelet adhesion in PrCa CTCs is proportionate to ovarian cancer cell lines (6.66%), then it was hypothesized that there was sufficient power to detect a difference of 2.65% with 100 participants in both exposed and non-exposed groups, determined by independent t-testing. A SD varying from 2% to 10% would enable detection of a difference of platelet cloaking of between 0.79% and 3.9% [11]. With regard to the detection of changes in platelet cloaking with time, and taking the same assumptions regarding SD of platelet adhesion in PrCa CTCs, a change of 1.8% platelet cloaking between any two time points in the 100 participants in each of the exercise and the control groups will be detectable, determined by paired t testing [11]. The aforementioned SD from 2% to 10% would enable the detection of a difference in platelet cloaking of between 0.56% and 2.8% [11]. Clinical and demographic baseline characteristics were determined using descriptive statistics. For analysis of CTC number (S1 Table), a generalized linear mixed model with a negative binomial response was fit to the individual CTC counts with additive fixed effects for time, location (London n = 34, Ireland n = 27), a time-by-location interaction, and a person random effect to account for the correlation between measurements on the same person. All three fixed effects were statistically significant; therefore, CTC counts changed significantly through time and the effect of time on CTC counts varied by location. Locations were not analysed separately, as described, an effect for location was estimated, however all data were used to build one model for each response of interest to minimise bias. Therapy was not an inclusion/exclusion criterion for the trial, however treatment effect was also examined between the Irish and London cohorts. Missing data were removed from the datasets before analysis reducing the degrees of freedom in the analysis but not affecting the parameter estimates. A Bonferonni corrected p-value threshold of 0.05 was used to assess the statistical significance of the clinical variables while controlling the type I error rate. Logistic regression was used to model both binary responses platelet cloaking and CTC cluster analysis (1—presence/0—absence). Therefore, the probability that an individual in each group at each time point would provide a blood sample with evidence of platelet cloaking was estimated. Separately, participants were stratified based on high (≥ 25) or low (< 25) BMI and associations with clinical variables were correlated using simple linear regression. Multiple testing corrections and model validity assessments were performed to account for the large number of comparisons made throughout the analysis. Statistical analysis was performed using R version 3.4.0 (www.r-project.org).

## Results

### ExPeCT participant characteristics

**Fig 1:**
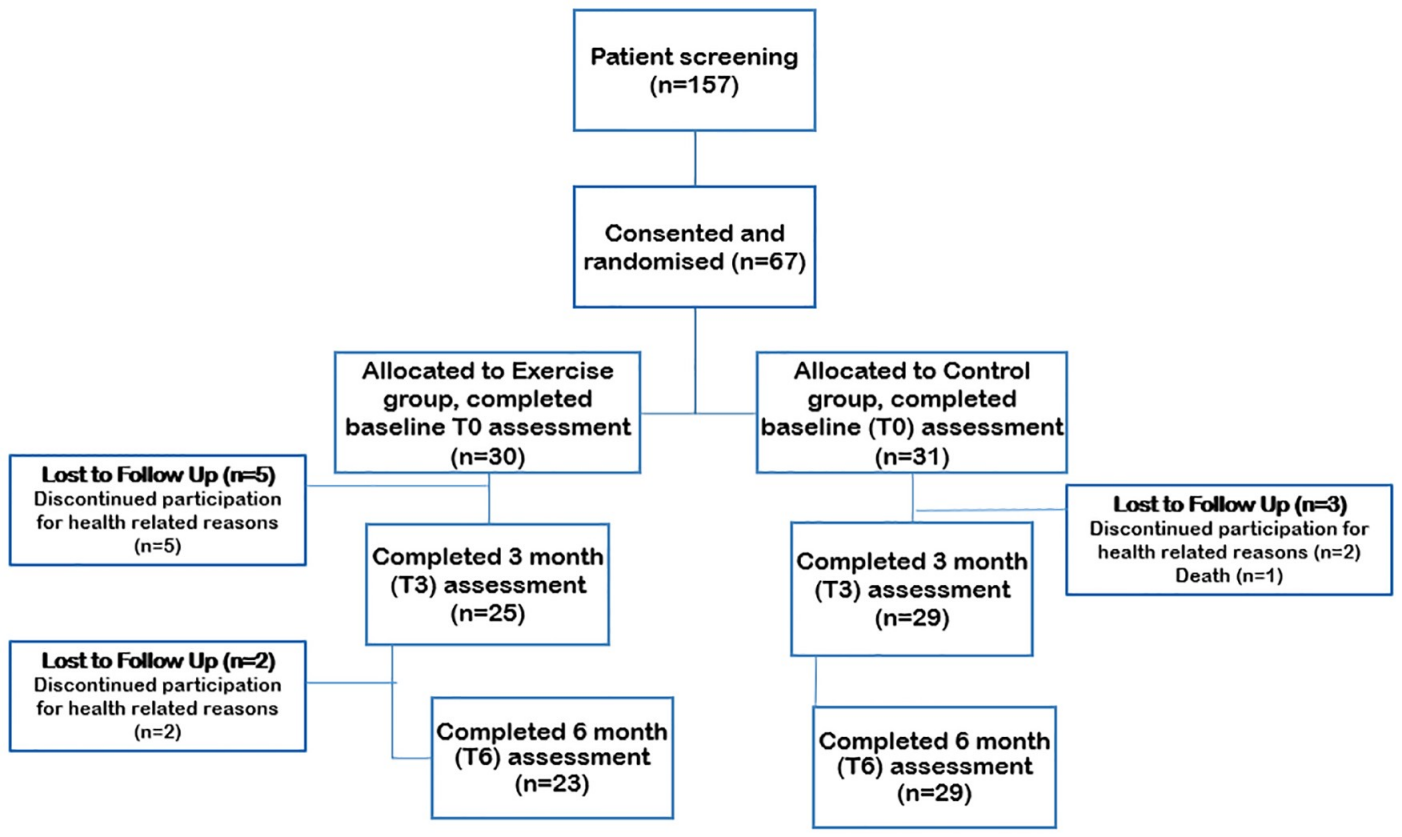
Flowchart of ExPeCT participant involvement.

The ExPeCT trial actively enrolled from 2014–2017. A schematic of participant numbers and groups are given in Fig 1. The exercise and control groups were similarly matched at baseline in terms of mean age (69 years), waist circumference (exercise 104 cm; control 100 cm) and median PSA (exercise 1.89 ng/mL; control 0.89 ng/mL) (Table 1). The majority of patients had bony metastases (exercise n = 27; control n = 28), with the remainder having lymph node and visceral metastases. Androgen Deprivation Therapy (ADT) was the most common treatment at baseline with the remainder receiving second generation anti-androgens, combination ADT and second generation anti-androgens, and/or chemotherapy (S2 Table). The exercise programme was well tolerated. Adherence was 83% for supervised exercise; 72% for the first three months of unsupervised home exercise and 67% in the last three months. No adverse events related to the exercise intervention were reported during the study.

**Table 1:** ExPeCT participant clinical and demographic characteristics at baseline.

	Control (n = 31)	Exercise (n = 30)
**Age, mean (SD)**	69.9 (7.5)	69.8 (7.0)
**BMI, n (%)**		
**<25**	3 (9.7)	8 (26.7)
**25–30**	14 (45.2)	11 (36.7)
**≥30**	14 (45.2)	11 (36.7)
**Waist circumference (cm), mean (SD)**	104.1 (11.7)	100.5 (14.6)
**Haemoglobin (g/L), mean (SD)**	126.1 (17.1)	131.9 (14.1)
**White cell count (cells/mm^3^), mean (SD)**	6.3 (5.2–10.1)	6.7 (5.1–8.4)
**Months since prostate cancer diagnosis, median (IQR)**	11.5 (7.0–48.0)	26.5 (8.0–63.0)
**Gleason score at diagnosis, n (%)**		
**7**	4 (14.3)	3 (12.0)
**8**	9 (32.1)	11 (44.0)
**9**	15 (53.6)	11 (44.0)
**missing**	3	5
**PSA (ng/mL) at T0, median (IQR)**	0.8 (0.1–10.00)	1.9 (0.2–11.1)

### Significant changes in CTC number observed over time in ExPeCT participants

**Fig 2:**
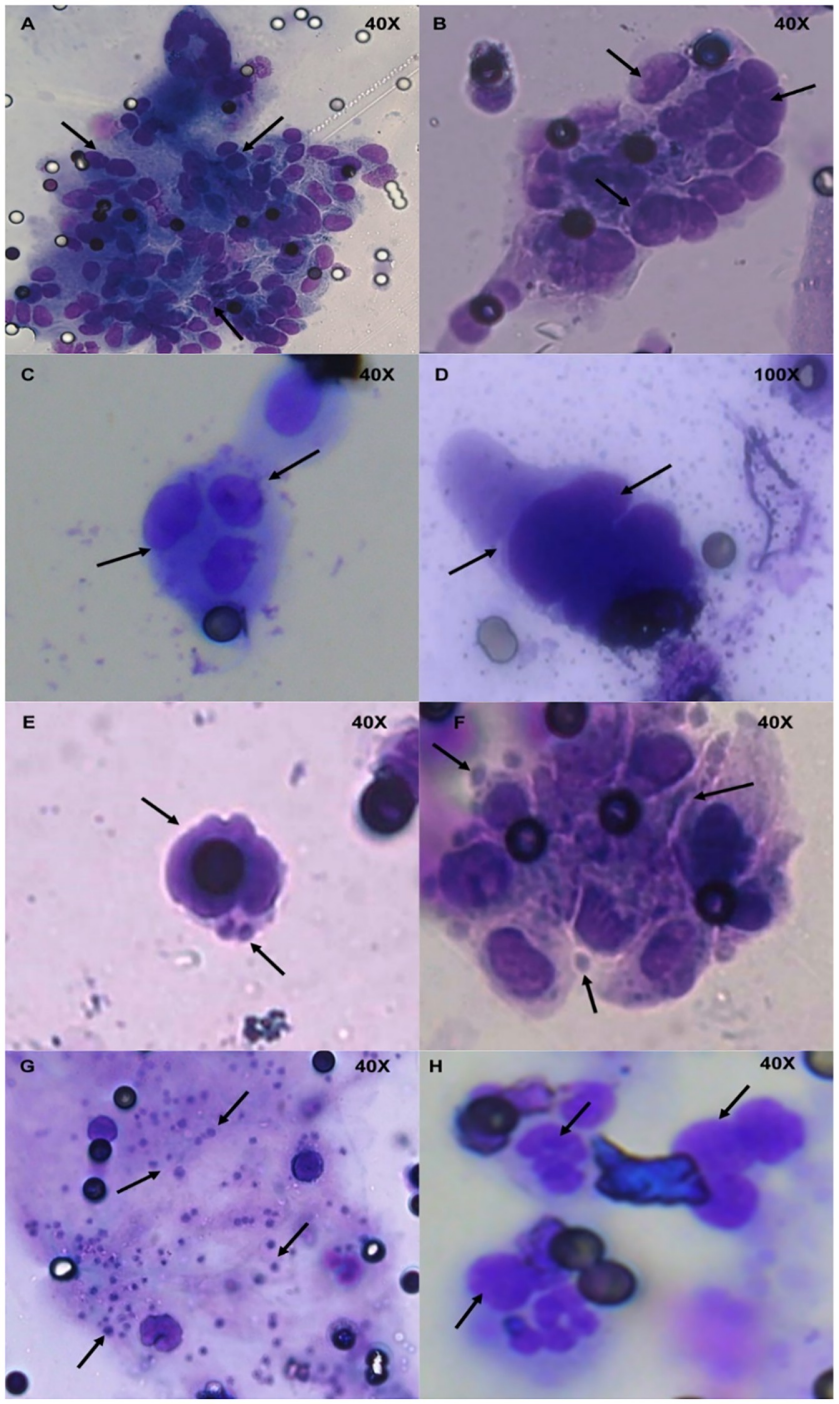
MGG morphological staining of representative ExPeCT filters. A) and B) arrows depict large groups of putative CTCs (CTC clusters) and filter pore, C) arrows indicate a group of four putative CTCs, D) arrows illustrate multiple CTCs without cytoplasm attached, E) and F) arrows depict suggested platelet cloaking of CTCs, G) arrows represent dispersed single platelets on a filter and H) arrows detail inflammatory cells present in a separate plane of focus. All images were taken using a 20x or 40x objective lens, filters pores measure 7.5 μm in diameter. MGG–May Grunwald Giemsa, CTC–circulating tumour cell.

**Fig 3:**
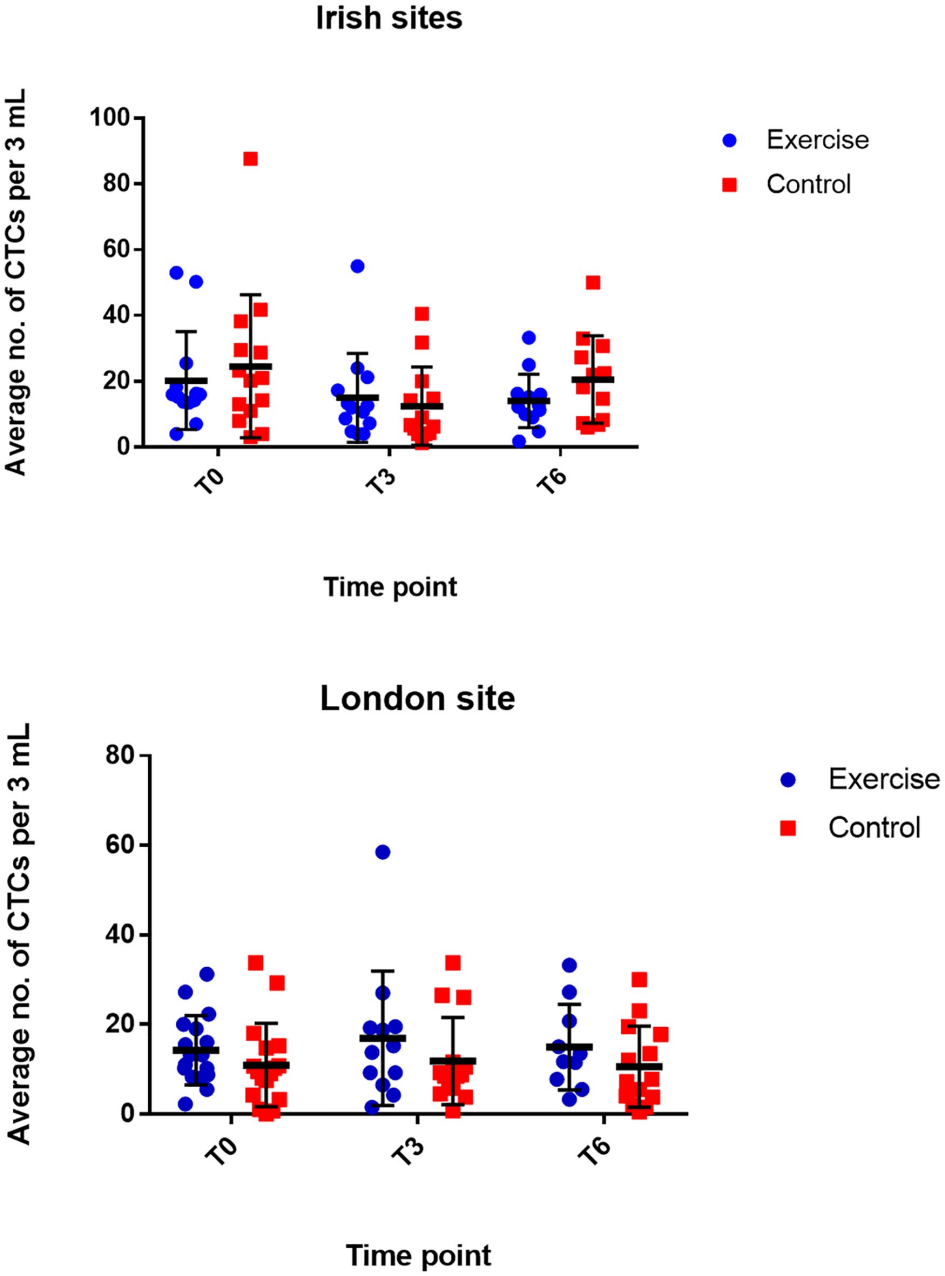
Average CTC count in 3 mL blood over time, exercise *vs*. control, at Irish and London sites. Location is demonstrated separately as there is a statistically significant difference between the sites. α—significant decrease in CTC number in exercise and control groups between T0 and T3, Irish cohort (p = 0.00); β–significant decrease in CTC number in exercise and control groups between T0 and T6, Irish cohort (p = 0.00); δ—significant decrease in CTC number in exercise and control groups between T0 and T6, London cohort (p = 0.00); λ—significant decrease in CTC number in exercise and control groups between T3 and T6, London cohort (p = 0.00); σ—significant decrease in CTC number in exercise and control groups between T0 and T6, London cohort (p = 0.00). Data graphed as mean ± SD. Exercise–Ireland n = 13, London n = 17 T0; Ireland n = 13, London n = 12 T3; Ireland n = 13, London n = 10 T6; Control–Ireland n = 14, London n = 17 T0; Ireland n = 13, London n = 15 T3; Ireland n = 12, London n = 15 T6. CTC–circulating tumour cell, T0 –baseline, T3 –three months, T6 six months, SD–standard deviation.

Morphological analysis, based on clinical cytology criteria, identified the presence of CTCs in all participant samples. CTC clusters were identified in 34% of participants across all time points (Fig 2). No significant change in CTC number over time between the two groups (p = 0.2630) was observed (Fig 3). However, significant decreases in CTC number over time were noted within each group (S3 Table).

### Platelet cloaking and CTC clusters identified in ExPeCT participants

To determine differences *in vitro* between platelet cloaking in normal *vs* malignant prostate cells, platelets and prostate cell lines DU145 and RWPE1 with epithelial morphology were spiked into healthy donor blood and processed according to ScreenCell guidelines. Filters were stained with MGG and analysed by a pathologist using clinically accepted cytology-based morphological criteria for the presence of platelet cloaking. Normal prostate cells displayed reduced cloaking compared to tumour cells (p = 0.0571) (S1 Fig). Additional validation experiments to determine isolation efficiency of the ScreenCell filtration method for both normal and malignant prostate cells are required but are beyond the scope of this current study. Platelet cloaking was identified based on the presence/absence of platelets adhered to identified CTCs at T0, T3 and T6 (Fig 2). Platelet cloaking was present in 29.5% of participants across all time points. A trend towards reduced platelet cloaking in the exercise group, when compared to the control group, over time (p = 0.1005) was observed. A lower number of CTC clusters were observed in the exercise group, when compared to the control group (p = 0.1663).

### Correlation of CTCs, platelet cloaking and CTC clusters to clinical variables

Standard clinical variables were examined for any relationship between CTCs, platelet cloaking and CTC clusters. There was no significant correlation observed between groups for CTCs, platelet cloaking or CTC clusters (Table 2). However, within both groups, a positive correlation was identified between CTCs and WCC (p = 0.0001) as well as CTC clusters and PSA level (p = 0.0393).

**Table 2:** Correlation between clinical variables and CTCs, clinical variables and platelet cloaking, and clinical variables and CTC clusters.

	Estimate	SD	z_value	p_value
**Average CTCs per 3 mL**				
**BMI**	0.01	1.58	0.00	1.00
**Weight**	0.97	1.52	0.64	0.52
**Height**	-1.11	1.23	-0.91	0.36
**Hb**	-0.50	1.35	-0.37	0.71
**WCC**	6.25	1.06	5.90	**0.00**
**PSA**	-1.03	1.18	-0.87	0.38
**WC**	1.10	1.45	0.76	0.45
**Platelet Cloaking**				
**BMI**	0.34	0.29	1.16	0.25
**Weight**	-0.08	0.32	-0.26	0.79
**Height**	-0.24	0.23	-1.02	0.31
**Hb**	-0.31	0.35	-0.89	0.37
**WCC**	-0.56	0.56	-1.02	0.31
**PSA**	0.07	0.24	0.30	0.76
**WC**	0.18	0.30	0.59	0.56
**CTC Clusters**				
**BMI**	-0.36	0.29	-1.23	0.22
**Weight**	-0.34	0.28	-1.20	0.23
**Height**	-0.42	0.30	-1.40	0.16
**Hb**	-0.14	0.30	-0.46	0.65
**WCC**	-0.22	0.36	-0.60	0.55
**PSA**	1.58	0.77	2.06	**0.04**
**WC**	-0.44	0.29	-1.55	0.12

Statistical analyses included a generalized mixed linear model (CTCs) and logistic regression (platelet cloaking and CTC clusters). Bonferonni corrected p-value threshold of 0.05.

BMI body mass index, Hb hemoglobin, PSA prostate specific antigen, SD standard deviation, WC waist circumference, WCC white cell count.

### High BMI is associated with higher WCC

At T0, all ExPeCT participants were divided into two groups based on their BMI, irrespective of their randomised trial arm (normal weight: BMI<25; n = 11 and overweight/obese: BMI≥25; n = 50). No participants presented as underweight (BMI<18). The mean number of CTCs and the presence of platelet cloaking at baseline was compared between BMI groups, with no significant difference noted (p = 0.88 and p = 0.785, respectively). A linear regression model was fitted to correlate baseline clinical variables with BMI (Table 3). High BMI was associated with higher WCC (p = 0.0001).

**Table 3:** Correlation between BMI and clinical variables.

Variable	Mean	SD	Regression Parameter (ƴ)	p-value
**BMI < 25**				
**Weight**	69.63	6.64	2.75	0.51
**Height**	175.61	8.55	4.13	0.25
**Hb**	128.78	11.41	0.40	0.93
**WCC**	7.64	3.07	0.56	0.90
**PSA**	18.49	32.13	-3.46	0.35
**WCir**	7.64	3.07	0.56	0.90
**BMI ≥ 25**				
**Weight**	89.39	12.01	0.83	0.72
**Height**	167.81	25.55	-2.44	0.29
**Hb**	129.04	16.80	-1.38	0.56
**WCC**	7.76	5.33	10.63	**0.00**
**PSA**	48.42	210.78	-2.12	0.36
**WCir**	105.43	11.26	2.62	0.26

Linear regression model. Bonferonni corrected p-value threshold of 0.05.

BMI body mass index, Hb hemoglobin, PSA prostate specific antigen, SD standard deviation, WC waist circumference, WCC white cell count.

## Discussion

The ExPeCT trial demonstrated high adherence from participants with metastatic PrCa to participate safely and effectively in a structured exercise programme. The primary endpoint was to examine the impact of exercise on CTC number and platelet cloaking and it was the first trial of its kind to do so. While the impact of exercise on CTC numbers has not been documented previously, no distinct difference was observed between the exercise and control cohorts, with respect to CTC number over time. ExPeCT was comprised solely of an aerobic exercise intervention, however, it is possible that a combination of aerobic and resistance exercises may have a more profound effect on CTC number. Previous and ongoing trials in men with metastatic PrCa have incorporated resistance elements [19–21], however none have examined the effect on CTCs.

CTCs were assessed morphologically and generally lacked cytoplasm and were represented by intact CTCs or bare CTC nuclei. It is possible that the process of filtration may have stripped CTCs of their cytoplasm. Morphological diagnostic criteria for CTCs are not well established and may vary between tumour types [22, 23]. In a study of patients with non-small cell lung cancer [24], cells meeting at least two of the following criteria were defined as circulating malignant cells, and as uncertain malignant cells when less than two criteria were present: anisonucleosis (ratio > 0.5), nuclei larger than three PD (i.e. > 24 μm), irregular nuclei, high nuclear:cytoplasmic ratio, presence of three-dimensional cell groups. Cells without visible cytoplasm were not included in the study. Among 10 cyto-pathologists there was excellent interobserver agreement for the detection of circulating malignant cells. Future work validating the morphologic criteria outlined in this study with a parallel CTC assay would aid in furthering defining these cells. In Irish sites there was a significant decrease in CTC number in both the control and exercise arms between T0 and T3, with an increase in CTC number within group observed between T3 and T6. The London site demonstrated a significant decrease in CTC number within group over time. Specific therapeutic use was not an exclusion criterion for ExPeCT therefore changes in CTC number may be due in part to the systemic therapy of each participant. A larger number of participants in the UK cohort were undergoing treatment with second-generation anti-androgens when compared to the Irish cohort, who had a higher prevalence of ADT. However, the inclusion of treatment data into the statistical model did not identify a treatment-location effect.

The identification of CTC clusters was rare, but CTCs within clusters usually retained their cytoplasm. CTCs within CTC clusters have demonstrated potential for increased survival and reduced levels of apoptosis, demonstrating greater capacity for forming metastasis [25]. The observed presence of CTC clusters was positively correlated with PSA levels. CTC clusters are associated with aggressive disease sub types in other cancers [26] and PSA is considered an independent marker for aggressive PrCa [27]. Little is known about CTC clusters in metastatic PrCa and taken together, the presence of CTC clusters and high PSA, may hold potential as a robust screening tool to identify more aggressive disease.

Platelet cloaking was present at a lower level than anticipated. It is likely that the relative rarity of platelet cloaking is due to the aforementioned artefactual removal of the cytoplasm during filtration, which has proven to be a significant limitation of this morphological size-based technique for the identification of CTC platelet cloaking. The interactions between CTCs and platelets are complex [28], however, overall tumour cell induced platelet aggregation correlates with metastatic potential and may be due to “cloaking’ of tumour cells by adherent platelets. Where platelet cloaking was identified it was confined to CTCs which had retained their cytoplasm. A trend associated with increased platelet cloaking was discernible in the control group, when compared to the exercise group. The exercise intervention did not have a significant impact on the presence of platelet cloaking over time, however the likelihood of platelet cloaking being present was greater within the control group. There were no significant relationships between platelet cloaking and clinical data.

Interestingly, a positive correlation between CTC number and WCC was recorded within group, for the first time in PrCa. A number of studies investigating the relationship between WCC and tumour cells have previously determined possible crosstalk between tumour cells and the immune response, with the potential for an immune evasion mechanism. Further investigation is required to improve our understanding of these novel data.

Obesity in advanced PrCa is correlated with worse outcomes [29]. Therefore, participants were sub-divided within each group depending on their BMI, and their BMI status compared to clinical variables and CTC counts. No significant differences were observed between BMI groups, in terms of platelet cloaking and CTC number. CTC number and BMI has previously been investigated in breast cancer, with BMI not independently associated with CTC number [30]. The low number of patients within the normal weight range may be a factor impacting this specific study outcome. These numbers are likely reflective of this population [31, 32], in addition to the prevalence of central adiposity as a common side effect of ADT [33]. A significant association was noted between high BMI and WCC count. These findings may signify a role between obesity related mediators and blood cells, which has been identified previously [34–36]. These findings, when combined with the relationship between CTCs and WCC, may suggest a synergistic role in advanced disease.

A limitation of this research is the number of patients accrued. ExPeCT was initially powered based on the primary endpoint of CTCs and platelet cloaking for a total of 200 participants based on the estimated numbers of men living with advanced disease in Ireland [1]. Reasons for not participating in the trial included co-morbidities that rule out the ability to partake safely in an exercise intervention and reasons similar to those reported in previous clinical trials [37]. Secondly, the CTC isolation method was filtration based with subsequent morphological identification. This method may have excluded smaller CTCs present in the blood, in addition to contributing to an enhanced cytoplasmic shearing effect. Future studies utilizing different CTC capture methods and immunocytochemistry markers would provide validation of enumeration and CTC morphological criteria. Lastly, while, the metastatic prostate cancer population tend to be a heterogenous group, this resulted in additional limitation of this study. Inclusion criteria did not distinguish metastatic subtype, therefore no subgroup analyses could be performed based on disease progression or treatment response. The primary aim of this study was to examine the role of CTCs and platelet cloaking in response to exercise, and the control and exercise groups were equally heterogenous. Future studies, with a protocol which allows for broad collection of clinical data, may provide information to further underpin the relationship between exercise and the metastatic cascade. However, this study highlights the ability of patients with metastatic PrCa to engage in clinical trials of this nature and ExPeCT has provided the foundation for larger scale studies to further elucidate exercise as an effective adjunct to treatment for patients living with metastatic disease.

## Conclusion

Although the primary endpoint of this study was not reached, there were a number of notable novel findings. Our study is the first to detect platelet cloaking in patients with metastatic PrCa. Although collection of data was restricted to total WCC, we have discovered a significant relationship between CTC number and WCC. This is an interesting finding, primarily in the context of the importance of the neutrophil lymphocyte ratio, as a synergistic relationship may exist between WCC and CTCs in the promotion of metastatic disease [38]. Furthermore, we have determined a significant association between CTC clusters and PSA, thus indicating that clusters may be more important than single CTCs in the development of advanced PrCa. Overall this study provides a critical insight into the metastatic cascade in PrCa.

## Supporting information

S1 Table Averaged CTC counts for all patients, control and exercise. A maximum of four filters was averaged per patient (3 mL whole blood).
(DOCX)

S2 Table ExPeCT participant classification and alterations to treatment course. (DOCX)

S3 Table Changes in CTC number over time within study group. (DOCX)

S1 Fig Platelet cloaking in tumour and normal cells. (TIF)

S1 Checklist CONSORT 2010 checklist of information to include when reporting a randomised trial. (DOC)

S1 File The ExPeCT trial protocol. (PDF)

S2 File ExPeCT protocol version 1.4 (14-Sep-2015). (PDF)

## Abbreviations

ADT: Androgen deprivation therapy
BMI: Body mass index
CRPC: Castrate resistant prostate cancer
CTC: Circulating tumour cell
Hb: Haemoglobin
HR: Heart rate
HRR: Heart rate reserve
MGG: May Grunwald Giemsa
NK: Natural killer
OS: Overall survival
PrCa: Prostate Cancer
PSA: Prostate specific antigen
RT: Room temperature
SD: Standard deviation
T0: Baseline
T3: Three months
T6: Six months
UK: United Kingdom
WC: Waist circumference
WCC: White cell count
WCRF: World Cancer Research Fund
