# Supplementary material for: Platelet cloaking of circulating tumour cells in patients with metastatic prostate cancer: Results from ExPeCT, a randomised controlled trial
Source: PLoS One. 2020 Dec 18;15(12):e0243928. doi: 10.1371/journal.pone.0243928 (PMC7748139; doi:10.1371/journal.pone.0243928)
Supplement: S1 Table — A maximum of four filters was averaged per patient (3 mL whole blood). (DOCX) [file pone.0243928.s001.docx]

**S1 Table:** Averaged CTC counts for all patients, control and exercise. A maximum of four filters was averaged per patient (3 mL whole blood per filter).

| Identifier | Group | Avg CTC Count T0 | Avg CTC Count T3 | Avg CTC Count T6 |
| --- | --- | --- | --- | --- |
| 001 | Exercise | 3.00 | 7.30 | 15.00 |
| 002 | Control | 11.00 | 6.67 | 8.25 |
| 003 | Control | 4.00 | 5.67 | No Sample |
| 004 | Exercise | 25.50 | 10.75 | 1.75 |
| 005 | Exercise | 7.00 | 4.00 | 4.75 |
| 006 | Control | 20.30 | 1.25 | 18.30 |
| 007 | Control | 28.75 | 14.75 | 22.50 |
| 008 | Control | 21.00 | 9.00 | 6.75 |
| 009 | Exercise | 13.75 | 4.75 | 9.00 |
| 010 | Control | 3.00 | 4.00 | No sample |
| 011 | Exercise | 15.25 | 4.00 | 12.75 |
| 012 | Exercise | 18.25 | 17.25 | 10.00 |
| 013 | Exercise | 16.00 | 12.00 | 16.25 |
| 014 | Control | 41.75 | 31.75 | 50.00 |
| 015 | Exercise | 13.50 | 13.30 | 16.00 |
| 016 | Control | 8.00 | 6.30 | 7.30 |
| 017 | Control | 13.00 | 14.25 | 6.00 |
| 018 | Control | 29.50 | 40.50 | 30.75 |
| 019 | Control | 87.67 | No sample | 33.00 |
| 020 | Exercise | 53.00 | 24.00 | 15.30 |
| 021 | Control | 38.30 | 20.00 | 22.00 |
| 022 | Exercise | 16.30 | 12.67 | 12.30 |
| 023 | Exercise | 50.30 | 55.00 | 33.30 |
| 024 | Control | 14.30 | 4.00 | 14.67 |
| 025 | Control | 23.30 | 4.30 | 27.30 |
| 026 | Exercise | 14.30 | 21.30 | 11.30 |
| 027 | Exercise | 16.00 | 8.70 | 25.00 |
| 028 | Control | 1.00 | Discontinued | Discontinued |
| 029 | Control | 0 (1 filter) | 8.75 | 0.50 |
| 030 | Exercise | 8.75 | 18.75 | 11.50 |
| 031 | Control | 15.25 | 33.75 | 30.00 |
| 032 | Exercise | 8.00 | 4.25 | 13.50 |
| 033 | Control | 3.25 | 4.50 | 5.50 |
| 034 | Exercise | 10.25 | 19.25 | 15.00 |
| 035 | Exercise | 13.25 | 15.25 | 27.25 |
| 036 | Control | 8.00 | 3.75 | 1.50 |
| 037 | Control | 0.67 | 0.75 | 2.00 |
| 038 | Exercise | 16.00 | 19.50 | Discontinued |
| 039 | Exercise | 13.25 | 6.50 | Discontinued |
| 040 | Exercise | 22.30 | 9.25 | 5.50 |
| 041 | Control | 10.25 | 7.75 | 3.75 |
| 042 | Control | 33.75 | 26.00 | 17.75 |
| 043 | Control | 18.00 | 5.75 | 19.50 |
| 044 | Exercise | 31.25 | 1.50 | 20.75 |
| 045 | Control | 10.67 | Discontinued | Discontinued |
| 046 | Exercise | 5.50 | Discontinued | Discontinued |
| 047 | Control | 7.50 | 11.60 | 12.00 |
| 048 | Control | 4.25 | 10.50 | 4.00 |
| 049 | Exercise | 8.25 | Discontinued | Discontinued |
| 050 | Control | 14.75 | 8.50 | 23.00 |
| 051 | Exercise | 27.25 | 27.00 | 33.25 |
| 052 | Exercise | 2.25 | Discontinued | Discontinued |
| 053 | Control | 9.50 | Discontinued | Discontinued |
| 054 | Exercise | 19.00 | Discontinued | Discontinued |
| 055 | Exercise | 20.00 | 58.50 | 11.67 |
| 056 | Control | 9.00 | 8.50 | 7.25 |
| 057 | Exercise | 10.25 | 9.25 | 3.30 |
| 058 | Control | 10.75 | 26.50 | 13.50 |
| 059 | Control | 29.25 | 9.25 | 7.75 |
| 060 | Exercise | 11.00 | 13.75 | 7.75 |
| 061 | Exercise | 15.50 | Discontinued | Discontinued |

Avg Average
