# Supplementary material for: Platelet cloaking of circulating tumour cells in patients with metastatic prostate cancer: Results from ExPeCT, a randomised controlled trial
Source: PLoS One. 2020 Dec 18;15(12):e0243928. doi: 10.1371/journal.pone.0243928 (PMC7748139; doi:10.1371/journal.pone.0243928)
Supplement: S2 Table — (DOCX) [file pone.0243928.s002.docx]

**S2 Table**: ExPeCT participant classification and alterations to treatment course.

| ExPeCT ID | CRPC/HSPC at Baseline (T0) | Treatment course (T3) | Treatment course(T6) |
| --- | --- | --- | --- |
| 001 | CRPC | No change from T0 | No change from T3 |
| 002 | HSPC | No change from T0 | No change from T3 |
| 003 | HSPC | No change from T0 | No change from T3 |
| 004 | HSPC | Treatment change from T0 | No change from T3 |
| 005 | HSPC | Not recorded | Not recorded |
| 006 | HSPC | No change from T0 | No change from T3 |
| 007 | HSPC | No change from T0 | Treatment change from T3 |
| 008 | HSPC | No change from T0 | No change from T3 |
| 009 | HSPC | No change from T0 | No change from T3 |
| 010 | HSPC | Treatment change from T0 | Treatment change from T3 |
| 011 | HSPC | No change from T0 | No change from T3 |
| 012 | CRPC | No change from T0 | Treatment change from T3 |
| 013 | CRPC | No change from T0 | No change from T3 |
| 014 | CRPC | Treatment change from T0 | Treatment change from T3 |
| 015 | CRPC | No change from T0 | No change from T3 |
| 016 | HSPC | No change from T0 | Treatment change from T3 |
| 017 | CRPC | Treatment change from T0 | No change from T3 |
| 018 | CRPC | Treatment change from T0 | No change from T3 |
| 019 | CRPC | Treatment change from T0 | No Change from T3 |
| 020 | CRPC | No change from T0 | Not recorded |
| 021 | CRPC | No change from T0 | No change from T3 |
| 022 | CRPC | No change from T0 | No change from T3 |
| 023 | CRPC | Treatment change from T0 | No change from T3 |
| 024 | HSPC | No change from T0 | No change from T3 |
| 025 | HSPC | No change from T0 | No change from T3 |
| 026 | HSPC | No change from T0 | No change from T3 |
| 027 | HSPC | No change from T0 | No change from T3 |
| 028 | Not available | Discontinued | Discontinued |
| 029 | CRPC | No change from T0 | No change from T3 |
| 030 | CRPC | No change from T0 | No change from T3 |
| 031 | CRPC | No change from T0 | Not recorded |
| 032 | CRPC | Not recorded | Not recorded |
| 033 | HSPC | Not recorded | Treatment change from T3 |
| 034 | CRPC | No change from T0 | No change from T3 |
| 035 | HSPC | No change from T0 | No change from T3 |
| 036 | HSPC | No change from T0 | No change from T3 |
| 037 | HSPC | No change from T0 | No change from T3 |
| 038 | CRPC | No change from T0 | Discontinued |
| 039 | CRPC | Treatment change from T0 | Discontinued |
| 040 | CRPC | No change from T0 | Treatment change from T3 |
| 041 | CRPC | No change from T0 | No change from T3 |
| 042 | CRPC | Treatment change from T0 | Treatment change from T3 |
| 043 | CRPC | No change from T0 | No change from T3 |
| 044 | CRPC | Treatment change from T0 | No change from T3 |
| 045 | HSPC | Discontinued | Discontinued |
| 046 | CRPC | Discontinued | Discontinued |
| 047 | CRPC | Treatment change from T0 | No change from T3 |
| 048 | CRPC | No change from T0 | Treatment change from T3 |
| 049 | CRPC | Discontinued | Discontinued |
| 050 | CRPC | No change from T0 | No change from T3 |
| 051 | CRPC | No change from T0 | No change from T3 |
| 052 | CRPC | Discontinued | Discontinued |
| 053 | CRPC | Discontinued | Discontinued |
| 054 | CRPC | Discontinued | Discontinued |
| 055 | CRPC | No change from T0 | Treatment change from T3 |
| 056 | CRPC | No change from T0 | No change from T3 |
| 057 | CRPC | No change from T0 | No change from T3 |
| 058 | CRPC | No change from T0 | No change from T3 |
| 059 | CRPC | No change from T0 | No change from T3 |
| 060 | CRPC | No change from T0 | Treatment change from T3 |
| 061 | CRPC | Discontinued | Discontinued |
