# Supplementary material for: Platelet cloaking of circulating tumour cells in patients with metastatic prostate cancer: Results from ExPeCT, a randomised controlled trial
Source: PLoS One. 2020 Dec 18;15(12):e0243928. doi: 10.1371/journal.pone.0243928 (PMC7748139; doi:10.1371/journal.pone.0243928)
Supplement: S3 Table — (DOCX) [file pone.0243928.s003.docx]

**S3 Table:** Changes in CTC number between exercise and control groups, and within exercise and control groups.

|  |  | *Estimate* | *SD* | *z_value* | *p_value* |
| --- | --- | --- | --- | --- | --- |
| *Between study groups* | ***Exercise - Control*** | 0.70 | 2.77 | 0.25 | 0.80 |
| *Ireland (within study group)* | ***T3-T0*** | -7.60 | 2.38 | -3.19 | 0.00 |
|  | ***T6-T0*** | -6.10 | 2.41 | -2.52 | 0.01 |
|  | ***T6-T3*** | 1.50 | 2.41 | 0.62 | 0.53 |
| *London (within study group)* | ***T3-T0*** | -8.88 | 3.43 | -2.59 | 0.01 |
|  | ***T6-T0*** | -10.66 | 3.47 | -3.07 | 0.00 |
|  | ***T6-T3*** | -11.66 | 4.08 | -2.86 | 0.00 |

Generalized mixed linear model with person random effects to account for repeated measures structure. Bonferonni corrected p-value threshold of 0.05.

SD standard deviation
