# Supplementary figures and images for: Platelet cloaking of circulating tumour cells in patients with metastatic prostate cancer: Results from ExPeCT, a randomised controlled trial
Source: PLoS One. 2020 Dec 18;15(12):e0243928. doi: 10.1371/journal.pone.0243928 (PMC7748139; doi:10.1371/journal.pone.0243928)

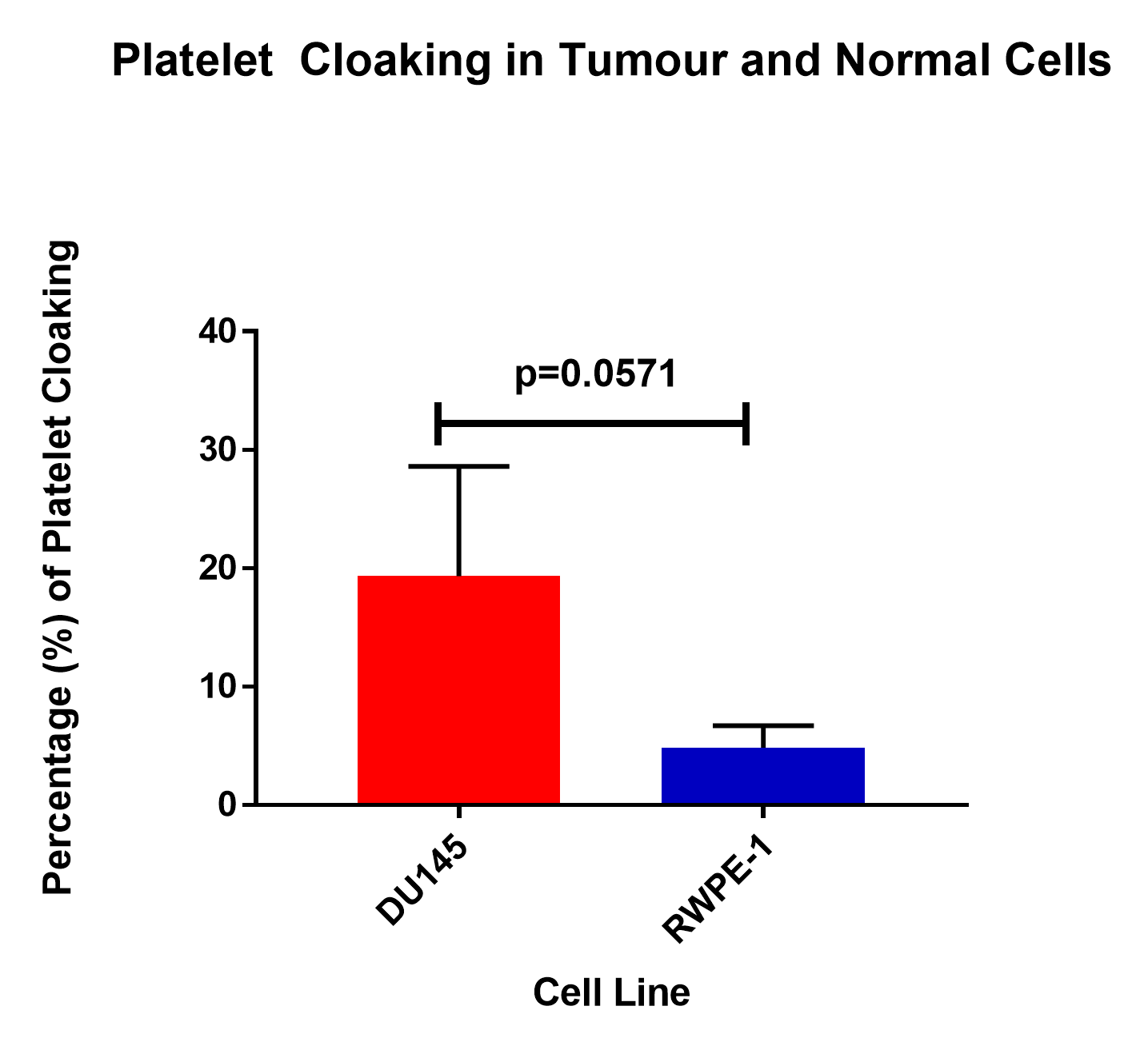

Supplement: S1 Fig — (TIF) [file pone.0243928.s004.tif]
