# Supplementary material for: Platelet cloaking of circulating tumour cells in patients with metastatic prostate cancer: Results from ExPeCT, a randomised controlled trial
Source: PLoS One. 2020 Dec 18;15(12):e0243928. doi: 10.1371/journal.pone.0243928 (PMC7748139; doi:10.1371/journal.pone.0243928)
Supplement: S1 File — (PDF) [file pone.0243928.s006.pdf]

Sheill et al. Trials (2017) 18:456
DOI 10.1186/s13063-017-2201-3

Trials

STUDY PROTOCOL Open Access

The ExPeCT (Examining Exercise, Prostate Cancer and Circulating Tumour Cells) trial: study protocol for a randomised controlled trial

Gráinne Sheill[1*†], Lauren Brady[2†], Emer Guinan[3], Brian Hayes[2,4], Orla Casey[5], John Greene[2], Tatjana Vlajnic[6], Fidelma Cahill[7], Mieke Van Hemelrijck[7], Nicola Peat[8], Sarah Rudman[7,8], Juliette Hussey[1], Moya Cunningham[5,9], Liam Grogan[5,10], Thomas Lynch[11], Rustom P. Manecksha[11], John McCaffrey[5,12], Lorelei Mucci[13], Orla Sheils[2], John O'Leary[2,14], Dearbhaile M. O'Donnell[5,15], Ray McDermott[5,16] and Stephen Finn[2,5,14]

Abstract

Background: Prostate cancer (PrCa) is the second most common cancer in Ireland. Many men present with locally advanced or metastatic cancer for whom curative surgery is inappropriate. Advanced cancer patients are encouraged to remain physically active and therefore there is a need to investigate how patients with metastatic disease tolerate physical activity programmes. Physical activity reduces levels of systemic inflammatory mediators and so an aerobic exercise intervention may represent an accessible and cost-effective means of ameliorating the pro-inflammatory effects of obesity and subsequently decrease poor cancer-specific outcomes in this patient population. This study will assess the feasibility and safety of introducing a structured aerobic exercise intervention to an advanced cancer population. This study will also examine if the evasion of immune editing by circulating tumour cells (CTCs) is an exercise-modifiable mechanism in obese men with prostate cancer.

Methods: This international multicentre prospective study will recruit men with metastatic prostate cancer. Participants will be recruited from centres in Dublin (Ireland) and London (UK). Participants will be divided into exposed and non-exposed groups based on body mass index (BMI) $\geq$ 25 kg/m^2 and randomised to intervention and control groups. The exercise group will undertake a regular supervised aerobic exercise programme, whereas the control group will not. Exercise intensity will be prescribed based on a target heart rate monitored by a polar heart rate monitor. Blood samples will be taken at recruitment and at 3 and 6 months to examine the primary endpoint of platelet cloaking of CTCs. Participants will complete a detailed questionnaire to assess quality of life (QoL) and other parameters at each visit.

Discussion: The overall aim of the ExPeCT trial is to examine the relationship between PrCa, exercise, obesity, and systemic inflammation, and to improve the overall QoL in men with advanced disease. Results will inform future work in this area examining biological markers of prognosis in advanced prostate cancer.

Trial registration: Clinicaltrials.gov NLM identifier: NCT02453139. Registered on 12 May 2015. This document contains excerpts from the ExPeCT trial protocol Version 1.5, 28 July 2016.

Keywords: Exercise, Advanced cancer, Metastatic, Prostate, Circulating tumour cells

* Correspondence: sheillg@tcd.ie
†Equal contributors
[1]Discipline of Physiotherapy, School of Medicine, Trinity College Dublin, Dublin, Ireland
Full list of author information is available at the end of the article

BioMed Central

© The Author(s). 2017 **Open Access** This article is distributed under the terms of the Creative Commons Attribution 4.0 International License (http://creativecommons.org/licenses/by/4.0/), which permits unrestricted use, distribution, and reproduction in any medium, provided you give appropriate credit to the original author(s) and the source, provide a link to the Creative Commons license, and indicate if changes were made. The Creative Commons Public Domain Dedication waiver (http://creativecommons.org/publicdomain/zero/1.0/) applies to the data made available in this article, unless otherwise stated.

Background

Prostate cancer

Prostate cancer (PrCa) is the most common cancer found in men in the developed world [1]. Many men present with locally advanced or metastatic cancer for whom curative surgery is inappropriate [2]. For these men, increases in progression-free and overall survival and quality of life (QoL) are the primary management objectives, and new therapies and assisting lifestyle alterations are increasingly needed.

Metabolic syndrome and prostate cancer

Obesity, known to be associated with a pro-inflammatory, pro-thrombotic humoral milieu, confers a worse prognosis in PrCa. Between 1990 and 2002, Irish male obesity increased from 8% to 20%, with a further 47% of men overweight [3]. Metabolic syndrome (MS) is a constellation of risk factors for cardiovascular disease, with central adiposity and insulin resistance being the most important components. Male hypogonadism, due to androgen deprivation therapy (ADT)—the mainstay of treatment for locally advanced and metastatic PrCa—is an independent risk factor for the various components of MS [4–8]. MS is present in 50% of all men undergoing long-term ADT [9] and is associated with progression of PrCa [10]. This may explain the excess non-cancer mortality in this population [11].

MS is characterised by low-level chronic systemic inflammation. Increasing evidence suggests that substantial cross-talk occurs between molecular pathways involved in inflammation, coagulation, and obesity [12]. Elucidation of how these pathways interact with PrCa cells may shed light on why obesity disimproves PrCa prognosis.

Circulating tumour cells and prostate cancer

Circulating tumour cells (CTCs) are identified in the blood in advanced cancer. Epithelial cells circulating in the blood of patients with carcinoma can be identified using various techniques including the ScreenCell® system (ScreenCell, Paris, France). Increasing evidence suggests that numbers of CTCs may have a prognostic role in advanced PrCa. A prospective study of castration-resistant PrCa found that ≥ 5 CTCs per 7.5 mL of blood correlated with a poor prognosis [13]. When a variety of clinical, serological, and pathological parameters were considered, the model best predictive of survival was based on baseline lactate dehydrogenase (LDH), baseline CTC count, and fold-change in CTC count at monthly intervals [14].

Natural killer cells and obesity

Natural killer (NK) cell numbers in blood and in solid organs, as well as NK cell cytotoxicity and cytokine secretion, are known to be reduced in obesity [15]. In addition, obese people with hypertension, raised fasting glucose, and an unfavourable lipid profile have less NK cells than "metabolically healthy" obese patients. Obese subjects have lower numbers of hepatic NK cells and leptin receptor-positive cells compared with those of normal weight [16]. The NK cell fraction of white blood cells is sensitive to exercise [17], and five-fold increases in NK concentrations following acute exercise have been noted. Brief exercise upregulates molecular pathways in circulating NK cells associated with cancer and cell communication [18]. In healthy young men, hypoxic exercise training leads to enhanced in-vitro NK cell cytotoxicity [19].

Interactions between platelets and circulating tumour cells

Despite the long-recognised association between cancer and thromboembolism, it has been unclear whether the thrombocytosis often seen in patients with metastases is a consequence or cause of widespread dissemination of the tumour. Accumulating evidence now shows that platelets support tumour metastasis by various mechanisms [20]. Platelets are involved in the arrest of CTCs in the vasculature and, through endothelial interactions, enable their extravasation. Platelets also secrete various pro-oncogenic factors including platelet-derived growth factor (PDGF) and vascular endothelial growth factor (VEGF), and mediate pro-survival signals in ovarian cancer cells [21].

Tumour cell-induced platelet aggregation correlates with metastatic potential, and may be due to "cloaking" of tumour cells by adherent platelets. The interaction between platelet cloaking of CTCs and tumour cell killing by NK cells is not completely understood. "Cloaking" of CTCs by adherent platelets may impede NK cell clearance of CTCs from the circulation, enhancing metastatic spread. Thrombocytopaenic mice exhibited reduced tumour metastatic burden when the tumour cells were NK cell sensitive, and in-vitro studies demonstrated reduced NK tumourilytic activity when platelets aggregated around tumour cells [22]. Platelets may enable evasion of immune editing by NK cells by conferring a "pseudonormal" phenotype on CTCs by encouraging high-level surface expression of normal major histocompatibility complex (MHC) class 1 antigen by the tumour cells [23].

In these pre-clinical studies there is an association between increased platelet-tumour cell interactions and endpoints of metastasis and death in animal models, but no clinical data exist as yet relating these interactions to outcomes in human disease. The current proposed study takes the current weight of evidence that platelet interactions are important in metastasis, and attempts to make the leap to demonstrate this in a clinical population. Platelet "cloaking" may be enhanced in obese

patients due to the pro-inflammatory, pro-thrombotic state, and may be a mechanism for worse cancer-specific outcomes in this group.

Prostate cancer and exercise

Several studies have shown that exercise may be protective against aggressive PrCa although there is no evidence that exercise protects against PrCa overall [24–27]. In PrCa patients there is solid evidence that exercise (especially group exercise) improves muscular and aerobic endurance, reduces fatigue, and improves overall quality of life [28].

Physical activity reduces levels of systemic inflammatory mediators [29], such as tumour necrosis factor (TNF)α, and so exercise may represent an accessible and cost-effective means of ameliorating the pro-inflammatory effects of obesity. This effect of physical activity depends on type, volume, and intensity, and does not depend directly on weight loss [30].

Obesity and its biochemical effects may be influenced by lifestyle changes such as exercise. As physical activity reduces levels of systemic inflammatory mediators, aerobic exercise may represent an accessible and cost-effective means of ameliorating the pro-inflammatory effects of obesity.

Methods and design

ExPeCT study objectives

The overarching hypothesis is that enhanced platelet cloaking of CTCs in obese men with prostate cancer, due to increased systemic inflammation, is a mechanism underlying worse prognosis of cancer in these patients.

The aim is to test the following four hypotheses, dividing the experimental and analytical work into four separate projects:

1. Platelet cloaking of circulating PrCa tumour cells is more prominent in men with obesity than without.
2. Regular exercise can ameliorate platelet cloaking.
3. The degree of platelet cloaking varies with levels of systemic and primary tumour inflammation and coagulability.
4. Expression of an obesity-associated lethality gene signature leads to variation in platelet cloaking.

ExPeCT study design

This international multicentre prospective study will recruit men with metastatic PrCa from five Irish hospitals and one UK hospital. This study incorporates both an observational component, with exposed and non-exposed groups defined based on body mass index (BMI), and an exercise component, with randomization to exercise and control groups for a supervised exercise programme. Participants with metastatic prostate cancer will be recruited and divided into exposed ($BMI \geq 25$ kg/m^2) and non-exposed groups ($BMI < 25$ kg/m^2). All exposed and non-exposed participants will be randomised to an exercise group or a control group, helping to minimise bias. The exercise group will participate in a 6-month exercise programme, comprising a weekly group exercise class and a home-based exercise programme. Participants will also be encouraged to complete activity diaries. From baseline (T0) to 3 months (T3), participants in the exercise arm will meet in small groups with a chartered physiotherapist for 1 h per week. At these sessions, participants will be educated about using the Polar heart rate monitors, prescribed their target exercise intensity, and complete a half-hour group aerobic exercise class. From T3 to 6 months (T6) continued aerobic exercise will be encouraged but classes will not be supervised by a chartered physiotherapist. All patients will be offered a personal exercise advice session at the study end to discuss long-term compliance to physical activity guidelines. Any patients demonstrating a need for further follow-up in relation to their physical activity levels will be advised to attend their general practitioner (GP) for a referral to the GP exercise scheme.

The study design consists of four main projects (Fig. 1):

Project 1: CTCs will be enumerated in the T0 samples. Adherent platelets will be quantified and compared between the exposed and non-exposed groups, and correlated with clinicopathological parameters.

Project 2: The exercise group will undertake a regular supervised aerobic exercise programme, whereas the control group will not. T3 and T6 blood samples will be assessed for CTC numbers and platelet cloaking. Changes will be compared with the T0 sample, and between exposed and non-exposed, and exercise and control groups. Participants will complete a detailed questionnaire to assess QoL and other parameters at each visit.

Project 3: Blood samples will be assessed for NK cell number and activation, markers of systemic inflammation, adipokines, and serum factors related to platelet activation. The prostate needle core biopsies (NCBs) will be examined microscopically for atrophy and inflammation by morphology and immunohistochemistry, with particular reference to NK cells. All variables will be correlated with platelet cloaking.

Project 4: NCBs will be assessed for expression of an obesity-associated lethality gene signature (whose genes are known to play a role in obesity or platelet aggregation and coagulation), and correlated with platelet cloaking of CTCs.

ExPeCT participant selection criteria

Inclusion criteria

1. Written informed consent obtained before any study-related procedures.

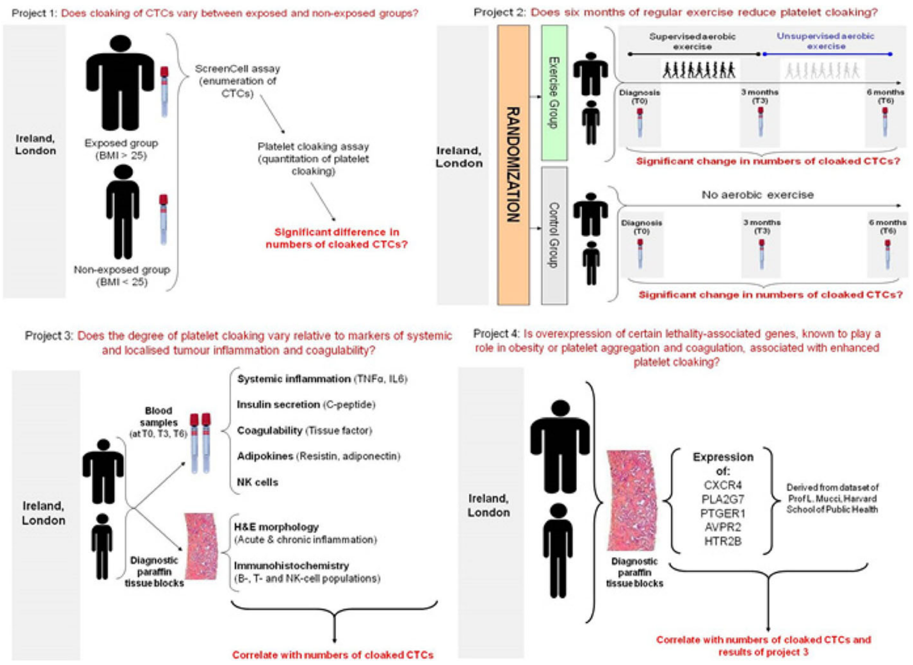

Fig. 1 Schematic of the four projects involved in the ExPeCT trial. *BMI* body mass index, *CTC* circulating tumour cell, *H&E* haematoxylin and eosin, *NK* natural killer, *TNF* tumour necrosis factor

2. Aged ≥ 18 years and male.
3. Histologically confirmed diagnosis of prostate adenocarcinoma.
4. M1 metastatic disease as confirmed by computed tomography (CT)/magnetic resonance imaging (MRI) or by bone scan, excluding patients who only have nodal metastatic disease.
5. Stable medical condition, including the absence of acute exacerbations of chronic illnesses, serious infections, or major surgery within 28 days prior to randomisation.
6. Capable of participating safely in the proposed exercise as assessed and signed off by a treating physician involved in ExPeCT recruitment.

Exclusion criteria

1. Patients with a history of radical prostatectomy.
2. Patients with other known malignancy (except non-melanoma skin cancers or fully excised carcinoma in situ at any site).

Participant enrolment procedure

Potential patients will be enrolled to the study on the basis of the inclusion/exclusion criteria. Enrolment of patients will be undertaken by staff at the medical oncology clinics at each recruiting site as well as members of the ExPeCT research team who have been delegated this task by the principle investigator (PI) (Fig. 2). Any queries about eligibility will be addressed directly to the Chief Investigator. Informed consent will be obtained by clinic staff or a member of the ExPeCT research team according to the requirements of International Conference on Harmonisation-Good Clinical Practice (ICH-GCP).

Upon registration of new participants, a signature confirming eligibility for the trial must be obtained from a treating physician involved in ExPeCT recruitment. Each registered patient will receive a unique participant identifier number (PIN). In order to ensure random allocation of participants to each study group, the computer programme Graphpad will be used to randomly assign a treatment group to each PIN. When issuing each PIN, two gatekeepers (1 in Ireland and 1 in the UK) will inform the research team of the treatment allocation of the participant. If a participant chooses to withdraw from the study, all data obtained up to the point of withdrawal will be carried forward unless requested otherwise.

Study methodology

Demographic and clinical characteristics

A datasheet will be completed for each participant after recruitment at T0 and at the T3 and T6 follow-up visits. Data gathered will include date of birth, anthropometric parameters (body weight, standing height, waist circumference), blood pressure, routine laboratory data (serum

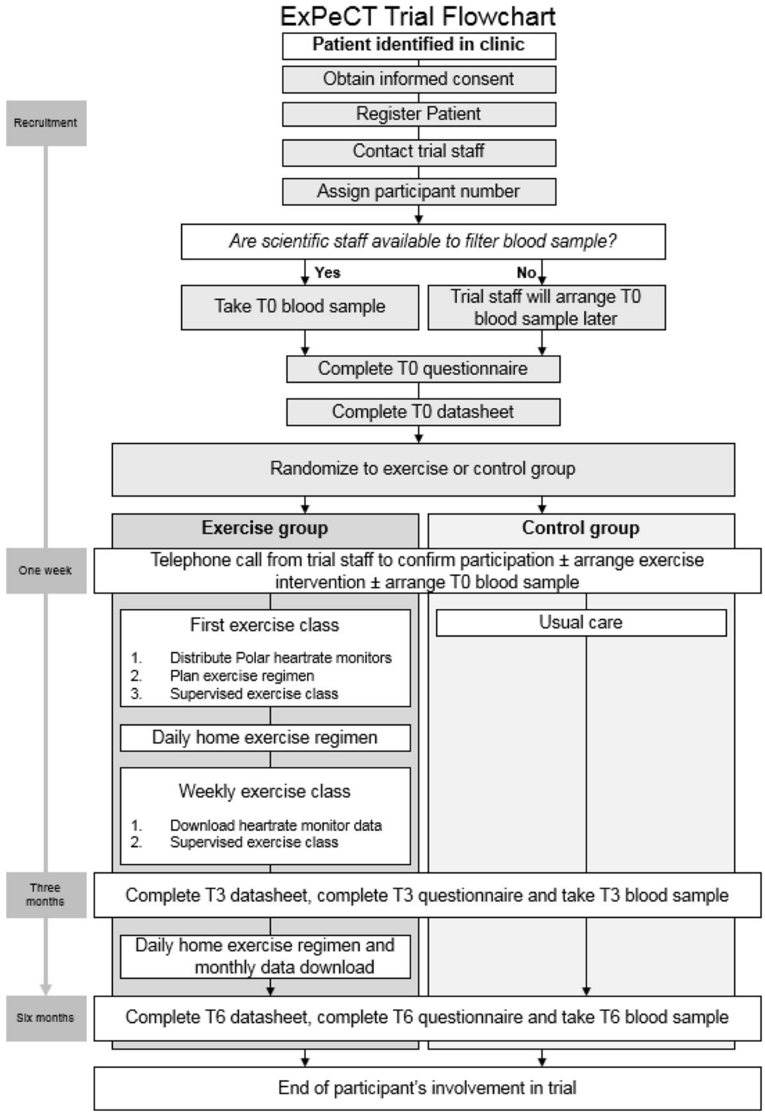

Fig. 2 ExPeCT trial flowsheet

prostate-specific antigen (PSA), haemoglobin, white cell and platelet counts), site of metastasis, and cancer-related data (stage and Gleason grade of cancer, details of current and previous systemic and radiation therapy). Data will also be recorded from three measures of physical function including balance, lower limb strength, and gait speed. These three measures will be completed with the patient by the chartered physiotherapist. Participants may also be asked to complete a structured interview session with the chartered physiotherapist exploring attitudes towards exercise. An overview of all data collected is included in Fig. 3.

Primary study endpoint

Platelet cloaking of circulating tumour cells

For each clinical review episode (at baseline and after 3 and 6 months), 12–16 mL of blood drawn from each patient into K_2-EDTA tubes will be filtered by a ScreenCell® Cyto kit within 4 h. CTC enrichment depends on vacuum-assisted filtration through a microporous membrane filter to separate CTCs from other blood cells on the basis of size. Three to five filters will be generated for each participant, two of which will be stained with May-Grunwald Giemsa, followed by a broad-spectrum epithelial marker, and one to three reserved for platelet cloaking assays and other relevant markers. CTCs will be enumerated cytologically. The degree of platelet adhesion to CTCs will be assessed by immunohistochemistry. The number of CTCs with adherent platelets will be counted, and the approximate number of platelets adherent to each cell will be estimated.

Type of Data	Details Collected	Instrument Used	Study Period: Enrolment	T0	T3	T6
		Eligibility Screen	X			
		Informed Consent	X			
		Allocation	X			
Demographic and Clinical Measures	Background Details (age at diagnosis, domiciliary situation, comorbidities, recent medications)	Physicians' Health Study Assessment		X	X	X
	Blood Pressure, BMI, Waist Circ.	Clinical Registration Form		X	X	X
Subjective Lifestyle Measures	Stress	Perceived Stress Scale – 4		X	X	X
	Memory and Cognition	Physicians' Health Study Assessment		X	X	X
	Pain	Brief Pain Inventory Scale		X	X	X
	Smoking and Alcohol	Physicians' Health Study Assessment		X	X	X
	Physical Activity	Physicians' Health Study Assessment		X	X	X
	Depression	PHQ-9		X	X	X
	Diet (dairy products, meat, vitamin D)	Physicians' Health Study Assessment		X	X	X
	Quality of Life	FACT-P		X	X	X
	Sleep	Pittsburgh Sleep Quality Index		X	X	X
Physical Function Measures	Physical Activity Measurements	Actigraph Accelerometers		X		
	Physical Performance Measurements	Short Physical Performance Battery		X	X	X
	Adherence to Exercise Programme	FT7 Polar Monitors, Patient Exercise Diaries		X	X	X
Biological Measures	Circulating Tumour Cells	ScreenCell® system		X	X	X
	Serum and Plasma	Vacuette® system		X	X	X
	Natural Killer Cells[a]	Vacuette® system		X	X	X
	Diagnostic Paraffin Tissue Blocks	Original diagnostic NCB paraffin tissue blocks	X			

Fig. 3 ExPeCT SPIRIT figure. [a]Natural killer cell testing will only be performed at Irish sites. *BMI* body mass index, *Circ.* Circumference, *FACT-P* Functional Assessment of Cancer Therapy scales for Men with Prostate Cancer, *NCB* needle core biopsy, *PHQ* Patient Health Questionnaire

Secondary study endpoints

Systemic and localized tumour inflammation and coagulability

This part of the project consists of measurement of systemic and prostate inflammation, markers of coagulation, cytokines, and NK cells. The substrates for this work will be blood samples taken from each participant at T0, T3, and T6, and the original diagnostic NCB paraffin tissue blocks. Examples of the serological and haematological tests include adiponectin, leptin, and resistin.

Expression of lethality-associated genes

This project will evaluate expression of selected genes known to be associated with PrCa progression, coagulation, and stem cell-like phenotype in diagnostic NCBs. Sections of formalin-fixed, paraffin-embedded tissue blocks will be cut from each patient's diagnostic prostate NCB specimen. These sections will be dissected by either laser capture microdissection or gross dissection. Ribonucleic acid (RNA) will be extracted from the microdissected tissue. Gene expression profiling will be undertaken on diagnostic biopsy material using custom-designed assays designed to detect only mRNA and to traverse the exonic junction. Assays for the genes CXCR4, PLA2G7, PTGER1, AVPR2, and HTR2B will be employed. Quantitation of results of polymerase chain reaction (PCR) will be undertaken using the $\Delta\Delta$Ct method, comparing the Ct values of the samples of interest with a control or calibrator such as a non-treated sample or RNA from normal tissue. Diagnostic material may be used for further gene expression analysis associated with obesity as part of the trial.

Quality of life assessment

All participants will complete a detailed questionnaire after recruitment at T0, and again at T3 and T6. The sections of the questionnaire are as follows:

1. Background details (age at diagnosis, domiciliary situation, comorbidities, recent medications).

2. Smoking and alcohol.
3. Sleep (Pittsburgh Sleep Quality Index [31]).
4. Stress (Perceived Stress Scale – 4).
5. Depression (Patient Health Questionnaire (PHQ)-9) [32].
6. Quality of life (FACT-P) [33].
7. Memory and cognition.
8. Physical activity.
9. Diet (dairy products, meat, vitamin D).
10. Pain (Brief Pain Inventory Scale) [34].

Some sections of the questionnaire are stand-alone validated instruments (such as the Functional Assessment of Cancer Therapy scales for Men with Prostate Cancer (FACT-P), which is designed to assess health-related quality of life in this setting [33]). Others, such as the sections on physical activity and diet, are derived from a prostate cancer-specific questionnaire used in the large Physicians' Health Study based at Harvard University [35].

Exercise programme

The exercise group will participate in a 6-month moderate-to-vigorous intensity aerobic exercise programme comprising a weekly class and a home-based aerobic exercise programme. Participants will also be encouraged to complete weekly activity diaries. From T0 to T3, participants in the exercise arm will meet in small groups with a chartered physiotherapist for 1 h per week. During the first class the participants will receive an introduction to the format of the exercise and will be educated on safe exercise practices and strategies to monitor exercise exertion.

Each exercise participant will receive, and be educated about using, a Polar heart rate monitor for the duration of the study. Participants will exercise to a prescribed heart rate range during class and home sessions. Exercise prescription will progress in intensity and duration during months 1 and 2 of the programme to reach the target 3 h per week (180 min/week) of moderate-to-vigorous intensity activity from month 3 onwards (Table 1). This level of activity has been previously shown to be associated with a 33% reduction in all-cause mortality following prostate cancer [36]. Participants will be encouraged to achieve this target exercise in six 30-min sessions throughout the week. However, flexibility will be allowed to facilitate longer or shorter session to a total of 180 min/week. Each exercise session must be of at least 10 min duration. The research team has previously shown that similar aerobic activity intensities can be achieved in cancer survivors through a home-based walking programme and that a Polar heart rate monitor was an acceptable means of monitoring activity intensity [37].

During months 1–3, data from the Polar heart rate monitor will be downloaded weekly to monitor adherence. Participants will be scheduled to attend the research centre once monthly from T3 to T6 to download data and encourage ongoing adherence to the programme. In addition, participants will receive weekly telephone contact from the ExPeCT research team from T3 to T6 to encourage adherence.

The control group will not be given specific advice regarding exercise beyond that considered usual medical care, and will not be invited to participate in the aerobic exercise group. Participants will be reviewed at T3 and T6 following the baseline visit and anthropometric measurements and further blood samples taken. Participants assigned to the control group will be offered a personal exercise advice session following completion of the T6 assessment.

Table 1 Exercise intensity during supervised classes

Supervised exercise classes		Exercise intensity (% heart rate reserve) by baseline fitness group			Duration
		Poor	Fair	Average	(min)
Month 1	Week 1	40–50%	50–60%	55–65%	20
	Week 2	40–50%	50–60%	55–65%	20
	Week 3	45–55%	55–65%	60–70%	20
	Week 4	45–55%	55–65%	60–70%	30
Month 2	Week 5	50–60%	60–70%	65–75%	30
	Week 6	50–60%	60–70%	65–75%	30
	Week 7	55–65%	65–75%	65–75%	30
	Week 8	55–65%	65–75%	65–75%	30
Month 3	Week 9	60–70%	65–75%	65–75%	30
	Week 10	60–70%	65–75%	65–75%	30
	Week 11	60–75%	65–75%	65–75%	30
	Week 12	60–75%	65–75%	65–75%	30

Exercise prescription

Participants will be asked to self-rate their baseline activity levels as one of three categories as per American College of Sports Medicine (ACSM) guidelines:

1) Sedentary or minimally active, not completing any moderate to vigorous activity (equivalent to poor fitness levels).
2) Sporadic physical activity, suboptimal exercise (equivalent to fair fitness levels).
3) Habitual physical activity, regular moderate to vigorous exercise (equivalent to average fitness levels).

Exercise intensity will be prescribed using individualised heart rate reserve (HRR) ranges in accordance with the ACSM guidelines. The following formula will be used to calculate HRR and heart rate (HR) range prescriptions: (target % × [maximum HR − resting HR] + resting HR). For each participant, age-predicted maximal HR will be calculated using the following equation: (206.9 − [0.67 × age]) [38]. Participants with self-rated 'poor' fitness levels (category 1) will commence the programme at an aerobic intensity of 40–50% HRR. Those with self-rated 'fair' fitness levels (category 2) will commence the programme at an aerobic intensity of 50–60% HRR, and those with self-rated 'average' fitness levels (category 3) will commence the programme at 55–65% HRR. The duration and frequency of the home exercise programme sessions is outlined in Table 2.

Patients will also be encouraged to use the Borg Breathlessness Scale. Using this scale, participants will give a subjective rating of perceived exertion. It is a widely used and reliable indicator to monitor and guide exercise intensity [39]. The scale allows individuals to subjectively rate their level of exertion during exercise and can be used to correlate exertion levels with exercise heart rates [40]. The Borg scale will be particularly valuable with participants on beta blockers as measures of exercise intensity are inaccurate or dampened on these medications and polar monitors may not reflect an accurate heart rate during exercise.

Forms of aerobic exercise undertaken at the supervised exercise classes will specifically avoid activities which may be associated with higher risk (e.g. the use of rowing machines in participants with lumbar spinal metastases). Walking on treadmills is a low-risk exercise activity.

Exercise follow-up

Participants will be invited to attend outpatient departments 6 months after T0 and the trial datasheet, questionnaire, and physical function measures will again be completed. Blood samples will be obtained in the same fashion as for the T0 visit. All patients will be offered a personal exercise advice session at study end to discuss long-term compliance to physical activity guidelines. Any patients demonstrating a need for further follow-up in relation to their physical activity levels will be advised to attend their GP for a referral to the GP exercise scheme. After this visit, participants will be thanked for their involvement and discharged from the study.

Table 2 Home-based exercise intensity

Home-based walking programme		Exercise intensity (% heart rate reserve) by baseline fitness group			Time	
		Poor	Fair	Average	Days/week	Duration (min)
Month 1	Week 1	40–50%	50–60%	55–65%	2	20
	Week 2	40–50%	50–60%	55–65%	3	20
	Week 3	45–55%	55–65%	60–70%	3	20
	Week 4	45–55%	55–65%	60–70%	3	30
Month 2	Week 5	50–60%	60–70%	65–75%	3	30
	Week 6	50–60%	60–70%	65–75%	4	30
	Week 7	55–65%	65–75%	65–75%	4	30
	Week 8	55–65%	65–75%	65–75%	5	30
Month 3	Week 9	60–70%	65–75%	65–75%	5	30
	Week 10	60–70%	65–75%	65–75%	5	30
	Week 11	60–75%	65–75%	65–75%	5	30
	Week 12	60–75%	65–75%	65–75%	5	30
Month 4	Weeks 13–16	60–75%	65–75%	65–75%	6	30
Month 5	Weeks 17–20	60–75%	65–75%	65–75%	6	30
Month 6	Weeks 12–24	60–75%	65–75%	65–75%	6	30

Study duration

The study is scheduled to last for 4 years; initial funding was drawn down in April 2014. Enrolment commenced in November 2014 and closed in May 2017 in order to allow enrolled participants to complete their 6 months of follow-up and exercise programme and for all laboratory work and analysis to be finished before the study completion date.

Patient withdrawal and off-study procedure

Patients are free to withdraw from participation in the study at any time upon request. An off-study form must be completed and sent to the ExPeCT research team if a patient withdraws from the study or leaves due to another reason (e.g. study completion, extraordinary medical circumstances, lost to follow-up).

Incident reporting

The occurrence and severity of any incidents from the time of consent to completion of the programme at 6 months will be recorded by the chartered physiotherapist on a standardised reporting form (e.g. adverse events occurring as a result of exercise or adverse reactions to study blood draws). All incidents will be reported to the site PI. Incidents will be followed until resolution or until a patient withdraws from the study or leaves due to another reason (e.g. study completion, extraordinary medical circumstances, lost to follow-up). Recurrent incidents in the same patient will be counted as separate incidents.

Data management

The ExPeCT research team will be the only people with access to the data collected in the course of this project. Data analysis will be performed at St. James's Hospital by the in-house bioinformatics team and other members of the ExPeCT research team. At the end of the study period, when all analysis is complete, data will be retained by the ExPeCT research team. Data will be securely stored for up to 10 years with the option of requesting ethical permission for a prolonged storage time.

Sample size

We will recruit 200 participants over the lifetime of the study, evenly divided between the exercise group and the control group. To calculate the power of the study, we used data from a previous study of ovarian cancer cell lines which showed approximately 2% platelet adhesion [21]. A standard deviation (SD) varying from 2% to 10% would enable us to detect a difference of platelet cloaking of between 0.79% and 3.9%. Research into this area is at an early stage and the clinical importance of specific incremental changes in the degree of platelet cloaking is as yet uncertain, but its elucidation is beyond the scope of this study.

With regard to the detection of changes in platelet cloaking with time, and taking the same assumptions regarding SD of platelet adhesion in PrCa CTCs as in project 1, we will be able to detect a change of 1.8% platelet cloaking between any two time points in the 100 participants in each of the exercise and the control groups, determined by paired *t* testing. A SD varying from 2% to 10% would enable us to detect a difference of platelet cloaking of between 0.56% and 2.8%. Generalised linear mixed models will be employed in order to account for the correlation between multiple measurements in the same experimental subject.

Statistical analysis

Project 1 will compare the number of cloaked platelets, comparing healthy weight and overweight men using either the *t* test or the non-parametric Mann-Whitney test, depending on the normality of the data. Linear regression models will be used to test the association between obesity and extent of platelet cloaking, adjusting for potential confounders such as age, use of medications, and smoking. If the data are not normally distributed then a log transformation will be employed. In addition to comparing overweight and healthy weight men as a binary exposure, BMI will be modelled as an ordinal variable (<18.5, 18.5–24.9, 25.0–27.4, 27.5–29.9, 30+) and as a continuous variable and to test for linear trends with the log likelihood ratio test of nested models.

Project 2 will compare measurements of platelet cloaking at baseline and months 3 and 6 follow-up time points among men randomised to the exercise and control arms, in both the exposed ($BMI \geq 25$) and non-exposed ($BMI < 25$) groups. Intention-to-treat analyses will use linear mixed-effect models to incorporate each biomarker for a given participant over time. BMI will also be stratified to look at potential effect modification. To estimate longitudinal changes in quality of life scores from baseline, the primary analysis will be carried out using a mixed-effects model for repeated measures.

Project 3 will examine the extent of the inflammatory infiltrate in diagnostic NCBs. All variables will be correlated with CTC numbers and platelet cloaking using basic descriptive statistics such as Pearson correlation coefficients for continuous variables and simple *t* tests for categorical variables. In the event of skewed distributions or sparse data, we will use non-parametric tests such as the Spearman correlation and Mann-Whitney. Moreover, a principal component analysis will be undertaken to estimate the proportion of variability in platelet cloaking and CTC number which is explained as a function of the obesity inflammatory biomarkers. The biomarkers will be modelled as principal components in the

linear regression and adjusted for potential confounders such as age, smoking, and other factors.

Project 4: Generalized linear regression models will be used to examine whether obesity is associated with expression of each of the five markers in the tumour tissue, adjusting for potential confounders such as age and smoking status, as well as clinical features. Obesity will be dichotomised as BMI greater or less than 25, and we will also model BMI as a continuous variable and examine tests for trend. The expression of each marker will be assessed with respect to the extent of platelet cloaking (high, intermediate, and low). The categorisation of platelet cloaking as high, intermediate, and low is dependent on the proportion of CTCs with adherent platelets (high > 75%, intermediate 25–75%, low < 25%). A gene score will be created by ranking individuals across expression of each gene in tertiles, assigning points for each marker as lowest tertile = 0, middle tertile = 1, upper tertile = 2, and calculating a summary score.

Ethics and research governance

The study protocol and other documentation have been approved by NRES Committee London—Camden & Islington (REC reference 14/LO/1859), The Mater Misericordia Hospital Research Ethics Committee, Dublin (REC reference: 1/378/1760), Beaumont Hospital Ethics (Medical Research) Committee, Dublin (REC Reference 15/73), SJH/AMNCH Research Ethics Committee, Dublin (REC Reference: 2014-11 List 41 (6)) and St Luke's Radiation Oncology Network, Dublin (REC Number not assigned. Trial referred to as ICORG 15-21 (sponsorship identifier)).

Cancer Trials Ireland is the sponsor for the Irish sites on this study (Protocol Number CTRIAL-IE (ICORG) 15-21).

Discussion

Many patients diagnosed with PrCa are not suitable for radical therapy because of the extent or grade of disease. In those patients who have potentially curable disease, obesity and its complications may make radical surgery impractical. ADT is itself a cause of obesity and metabolic syndrome. For all of these reasons, men with PrCa who are obese are less likely to be treated with curative intent. Medical therapy is improving for the cardiovascular complications of obesity which are the major competing cause of death in these men. As control of obesity-related cardiovascular risk factors improves, aggressiveness of PrCa becomes more important in determining the cause of mortality. It is known that obese men have a worse outlook regarding cancer-related mortality than non-obese men. The combination of an ageing population with an increased PrCa incidence, increasing obesity prevalence, and improved management of cardiovascular risk factors means that in the future, simply put, more men are going to die as a result of the deleterious effect of being overweight in advanced PrCa. Demonstration that platelet cloaking is a mechanism by which obesity disimproves PrCa survival would suggest that therapies targeted at points along the pathway of platelet activation could be efficacious. For example, adiponectin supplementation or blockade of interleukin (IL)-6 or TNFα could be useful. Comparison of the expression of lethality-associated genes between the primary site and CTCs could highlight genes which are upregulated as part of the metastatic pathway, with potential for targeted therapy.

ExPeCT aims to elucidate a potential mechanism by which obesity confers a worse prognosis in PrCa, two increasingly prevalent diseases in the Western world. ExPeCT hopes to show that a low-cost, accessible exercise programme can improve QoL and potentially ameliorate the effects of obesity through alterations in the systemic adipokine and inflammatory mediator profile.

Trial status

ExPeCT trial protocol Version 1.5, 28 July 2016. Recruitment was initiated in October 2014 and continued until May 2017. Data collection is ongoing for enrolled participants and is expected to conclude in November 2017.

Abbreviations

ACSM: American College of Sports Medicine; ADT: Androgen deprivation therapy; BMI: Body mass index; CT: Computed tomography; CTC: Circulating tumour cell; FACT-P: Functional Assessment of Cancer Therapy scales for Men with Prostate Cancer; GP: General practitioner; HR: Heart rate; HRR: Heart rate reserve; ICH-GCP: International Conference on Harmonisation-Good Clinical Practice; IL: Interleukin; K_2-EDTA: Ethylenediaminetetraacetic acid; LDH: Lactate dehydrogenase; MRI: Magnetic resonance imaging; MS: Metabolic syndrome; NCB: Needle core biopsy; NK: Natural killer; PCR: Polymerase chain reaction; PDGF: Platelet-derived growth factor; PHQ: Patient Health Questionnaire; PI: Principal Investigator; PIN: Participant identifier number; PrCa: Prostate cancer; PSA: Prostate-specific antigen; QoL: Quality of life; RNA: Ribonucleic acid; SD: Standard deviation; T0: Baseline; T3: Three months; T6: Six months; TNF: Tumour necrosis factor; VEGF: Vascular endothelial growth factor

Acknowledgements

The authors would like to acknowledge their partnership with the Transdisciplinary Prostate Cancer Partnership (ToPCaP; www.topcapteam.org).

Funding

The ExPeCT trial is funded by the World Cancer Research Fund, grant reference number: 2013/1003.

Availability of data and materials

Not applicable to this study.

Authors' contributions

All authors have read and approved the final manuscript. SF, DMOD, RMD, JH, BH, and JOL are co-applicants on the initial grant proposal. BH and EG contributed to the original grant proposal and protocol. GS and LB contributed to the production of this manuscript and to protocol development. OC contributed to study coordination.

Ethics approval and consent to participate
The study protocol and other documentation have been approved by NRES Committee London—Camden & Islington (REC reference 14/LO/1859), The Mater Misericordia Hospital Research Ethics Committee, Dublin (REC reference: 1/378/1760), Beaumont Hospital Ethics (Medical Research) Committee, Dublin (REC Reference 15/73), SJH/AMNCH Research Ethics Committee, Dublin (REC Reference: 2014-11 List 41 (6)) and St Luke's Radiation Oncology Network, Dublin (REC Number not assigned. Trial referred to as ICORG 15-21 (sponsorship identifier)). Written informed consent will be obtained from each participant before any study-related procedures.

Consent for publication
Not applicable to this study.

Competing interests
The authors declare that they have no competing interests.

Publisher's Note
Springer Nature remains neutral with regard to jurisdictional claims in published maps and institutional affiliations.

Author details
[1]Discipline of Physiotherapy, School of Medicine, Trinity College Dublin, Dublin, Ireland. [2]Department of Histopathology and Morbid Anatomy, Trinity Translational Medicine Institute, Dublin, Ireland. [3]School of Medicine, Trinity College Dublin, Dublin, Ireland. [4]Department of Histopathology, Cork University Hospital, Wilton, Cork, Ireland. [5]Cancer Trials Ireland, Dublin, Ireland. [6]Institute of Pathology, University Hospital Basel, Basel, Switzerland. [7]King's College London, School of Cancer and Pharmaceutical Sciences, Translational Oncology & Urology Research (TOUR) , London, UK. [8]Guy's and St Thomas' NHS Foundation Trust, London, UK. [9]Department of Radiation Oncology, St Luke's Hospital, Dublin, Ireland. [10]Department of Oncology, Beaumont Hospital, Dublin, Ireland. [11]Department of Urology, St James's Hospital, Dublin, Ireland. [12]Department of Oncology, Mater Misericordiae, Dublin, Ireland. [13]Department of Epidemiology, Harvard T.H. Chan School of Public Health, Boston, USA. [14]Department of Histopathology, St James's Hospital, Dublin, Ireland. [15]HOPE Directorate, St James's Hospital, Dublin, Ireland. [16]Department of Oncology, Adelaide and Meath Hospital incorporating the National Children's Hospital, Dublin, Ireland.

Received: 29 March 2017 Accepted: 6 September 2017
Published online: 04 October 2017

References
1. Ferlay J, et al. Estimates of worldwide burden of cancer in 2008: GLOBOCAN 2008. Int J Cancer. 2010;127(12):2893–917.
2. Jani AB. Management strategies for locally advanced prostate cancer. Drugs Aging. 2006;23(2):119–29.
3. McCarthy SN, Gibney MJ, Flynn A. Overweight, obesity and physical activity levels in Irish adults: evidence from the North/South Ireland food consumption survey. Proc Nutr Soc. 2002;61(1):3–7.
4. Smith JC, et al. The effects of induced hypogonadism on arterial stiffness, body composition, and metabolic parameters in males with prostate cancer. J Clin Endocrinol Metab. 2001;86(9):4261–7.
5. Dockery F, et al. Testosterone suppression in men with prostate cancer leads to an increase in arterial stiffness and hyperinsulinaemia. Clin Sci (Lond). 2003;104(2):195–201.
6. Smith MR, Lee H, Nathan DM. Insulin sensitivity during combined androgen blockade for prostate cancer. J Clin Endocrinol Metab. 2006;91(4):1305–8.
7. Keating NL, O'Malley AJ, Smith MR. Diabetes and cardiovascular disease during androgen deprivation therapy for prostate cancer. J Clin Oncol. 2006;24(27):4448–56.
8. Keating NL, et al. Diabetes and cardiovascular disease during androgen deprivation therapy: observational study of veterans with prostate cancer. J Natl Cancer Inst. 2010;102(1):39–46.
9. Braga-Basaria M, et al. Metabolic syndrome in men with prostate cancer undergoing long-term androgen-deprivation therapy. J Clin Oncol. 2006;24(24):3979–83.
10. Smith MR, et al. Diabetes and mortality in men with locally advanced prostate cancer: RTOG 92-02. J Clin Oncol. 2008;26(26):4333–9.
11. Van Hemelrijck M, et al. Absolute and relative risk of cardiovascular disease in men with prostate cancer: results from the population-based PCBaSe Sweden. J Clin Oncol. 2010;28(21):3448–56.
12. Zhang N, Lawrence DA. Tissue factor and obesity, a two-way street. Nat Med. 2011;17(11):1343–4.
13. de Bono JS, et al. Circulating tumor cells predict survival benefit from treatment in metastatic castration-resistant prostate cancer. Clin Cancer Res. 2008;14(19):6302–9.
14. Scher HI, et al. Circulating tumour cells as prognostic markers in progressive, castration-resistant prostate cancer: a reanalysis of IMMC38 trial data. Lancet Oncol. 2009;10(3):233–9.
15. Lynch LA, et al. Are natural killer cells protecting the metabolically healthy obese patient? Obesity (Silver Spring). 2009;17(3):601–5.
16. Lautenbach A, et al. Human obesity reduces the number of hepatic leptin receptor (ob-R) expressing NK cells. Endocr Res. 2011;36(4):158–66.
17. Timmons BW, Cieslak T. Human natural killer cell subsets and acute exercise: a brief review. Exerc Immunol Rev. 2008;14:8–23.
18. Radom-Aizik S, et al. Impact of brief exercise on peripheral blood NK cell gene and microRNA expression in young adults. J Appl Physiol (1985). 2013;114(5):628–36.
19. Wang JS, Weng TP. Hypoxic exercise training promotes antitumour cytotoxicity of natural killer cells in young men. Clin Sci (Lond). 2011;121(8):343–53.
20. Gay LJ, Felding-Habermann B. Contribution of platelets to tumour metastasis. Nat Rev Cancer. 2011;11(2):123–34.
21. Egan K, et al. Platelet adhesion and degranulation induce pro-survival and pro-angiogenic signalling in ovarian cancer cells. PLoS One. 2011;6(10):e26125.
22. Nieswandt B, et al. Lysis of tumor cells by natural killer cells in mice is impeded by platelets. Cancer Res. 1999;59(6):1295–300.
23. Placke T, et al. Platelet-derived MHC class I confers a pseudonormal phenotype to cancer cells that subverts the antitumor reactivity of natural killer immune cells. Cancer Res. 2012;72(2):440–8.
24. Giovannucci EL, et al. A prospective study of physical activity and incident and fatal prostate cancer. Arch Intern Med. 2005;165(9):1005–10.
25. Patel AV, et al. Recreational physical activity and risk of prostate cancer in a large cohort of US men. Cancer Epidemiol Biomarkers Prev. 2005;14(1):275–9.
26. Nilsen TI, Romundstad PR, Vatten LJ. Recreational physical activity and risk of prostate cancer: a prospective population-based study in Norway (the HUNT study). Int J Cancer. 2006;119(12):2943–7.
27. Johnsen NF, et al. Physical activity and risk of prostate cancer in the European Prospective Investigation into Cancer and Nutrition (EPIC) cohort. Int J Cancer. 2009;125(4):902–8.
28. Keogh JW, MacLeod RD. Body composition, physical fitness, functional performance, quality of life, and fatigue benefits of exercise for prostate cancer patients: a systematic review. J Pain Symptom Manage. 2012;43(1):96–110.
29. Ho SS, et al. Effects of chronic exercise training on inflammatory markers in Australian overweight and obese individuals in a randomized controlled trial. Inflammation. 2013;36(3):625–32.
30. Balducci S, et al. Anti-inflammatory effect of exercise training in subjects with type 2 diabetes and the metabolic syndrome is dependent on exercise modalities and independent of weight loss. Nutr Metab Cardiovasc Dis. 2010;20(8):608–17.
31. Buysse DJ, et al. The Pittsburgh Sleep Quality Index: a new instrument for psychiatric practice and research. Psychiatry Res. 1989;28(2):193–213.
32. Kroenke K, Spitzer RL, Williams JB. The PHQ-9: validity of a brief depression severity measure. J Gen Intern Med. 2001;16(9):606–13.
33. Esper P, et al. Measuring quality of life in men with prostate cancer using the functional assessment of cancer therapy-prostate instrument. Urology. 1997;50(6):920–8.
34. Cleeland CS, Ryan KM. Pain assessment: global use of the Brief Pain Inventory. Ann Acad Med Singapore. 1994;23(2):129–38.
35. Deo P, Nayak R, Rajpura J. Women's attitudes and health beliefs toward osteoporosis screening in a community pharmacy. J Osteoporos. 2013;2013:650136.
36. Kenfield SA, et al. Physical activity and survival after prostate cancer diagnosis in the health professionals follow-up study. J Clin Oncol. 2011;29(6):726–32.
37. Broderick JM, et al. Feasibility and efficacy of a supervised exercise intervention in de-conditioned cancer survivors during the early survivorship phase: the PEACH trial. J Cancer Surviv. 2013;7(4):551–62.

38. Kohl HW, et al. An empirical evaluation of the ACSM guidelines for exercise testing. Med Sci Sports Exerc. 1990;22(4):533–9.
39. Wilson RC, Jones PW. Long-term reproducibility of Borg scale estimates of breathlessness during exercise. Clin Sci (Lond). 1991;80(4):309–12.
40. Borg GA. Psychophysical bases of perceived exertion. Med Sci Sports Exerc. 1982;14(5):377–81.

Submit your next manuscript to BioMed Central and we will help you at every step:

- We accept pre-submission inquiries
- Our selector tool helps you to find the most relevant journal
- We provide round the clock customer support
- Convenient online submission
- Thorough peer review
- Inclusion in PubMed and all major indexing services
- Maximum visibility for your research

Submit your manuscript at
www.biomedcentral.com/submit

BioMed Central
