# Supplementary material for: Platelet cloaking of circulating tumour cells in patients with metastatic prostate cancer: Results from ExPeCT, a randomised controlled trial
Source: PLoS One. 2020 Dec 18;15(12):e0243928. doi: 10.1371/journal.pone.0243928 (PMC7748139; doi:10.1371/journal.pone.0243928)
Supplement: S2 File — (PDF) [file pone.0243928.s007.pdf]

Trial Protocol

The ExPeCT Trial
(Exercise, Prostate cancer and Circulating Tumour cells)

Evasion of immune editing by circulating tumour cells is an exercise-modifiable mechanism underlying aggressive behaviour in obese men with prostate cancer

Version Number
1.4

Version Date
14th September 2015

Authors
Dr Brian Hayes
Dr Emer Guinan

List of Amendments to date
Version 1.1 18th November 2014
Version 1.3 27th August 2015

ExPeCT Research Team

Chief Investigator
Dr Stephen Finn
Associate Professor, Department of Histopathology and Morbid Anatomy
Trinity College Dublin

Principal Investigators
Dr Dearbhaile O'Donnell
Consultant Medical Oncologist
St James's Hospital Dublin

Dr Ray McDermott
Consultant Medical Oncologist
Tallaght Hospital Dublin

Prof Liam Grogan
Consultant Medical Oncologist
Beaumont Hospital, Dublin

Dr Moya Cunningham
Consultant Radiation Oncologist
St. Luke's Centre at St James's Hospital, Dublin

Prof John McCaffrey
Consultant Medical Oncologist
Mater Misericordiae University Hospital, Dublin

Dr Sarah Rudman
Consultant Medical Oncologist
Guy's Hospital, London

Co-Investigators
Prof John O'Leary
Professor and Head of Department of Histopathology and Morbid Anatomy
Trinity College Dublin

Dr Juliette Hussey
Vice President Global Relations (TCD Global)
Trinity College Dublin

Dr Mieke Van Hemelrijck
Lecturer in Cancer Epidemiology
King's College, London

ExPeCT Research Team
Dr Brian Hayes
Consultant Histopathologist
Cork University Hospital

Dr Emer Guinan
Postdoctoral Fellow, Department of Physiotherapy
Trinity College Dublin

Dr Orla Casey
Postdoctoral Fellow, Department of Histopathology
Trinity College Dublin

Grainne Sheill
PhD Student, Department of Physiotherapy
Trinity College Dublin

Lauren Brady
PhD Student, Department of Histopathology
Trinity College Dublin

Dr Tatjana Vlajnic
Pathologist, Department of Histopathology
Trinity College Dublin/University Hospital of Basel

Dr John Greene
Medical Oncology Research Registrar
Trinity College Dublin/Tallaght Hospital

Carrie Burgess
Research Nurse
Guy’s Hospital, London

Fidelma Cahill
Research Nurse
Guy’s Hospital, London

Study Funder

World Cancer Research Fund (WCRF UK)

Study Sponsor

All Ireland Cooperative Clinical Research Group (ICORG)

Table of Contents:

Abbreviations 6
1. Abstract 7
1.1 Background 7
1.2 Objectives 7
1.3 Setting and Methods 7
1.4 Impact 8
2. Background and Rationale 8
2.1 Prostate cancer 8
2.2 Metabolic syndrome and prostate cancer 8
2.3 Circulating Tumour Cells and Prostate Cancer 8
2.4 Interactions between platelets and circulating tumour cells 9
2.5 Prostate cancer and exercise 10
2.6 Natural killer cells and obesity 10
2.7 Rationale for study 11
3. Study Objectives 12
4. Study Design 14
5. Number of Participants 14
6. Anticipated Enrolment Period 15
7. Duration of Patient Participation 15
8. Estimated Study Completion Date 15
9. Participant Selection Criteria 16
9.1 Inclusion criteria 16
9.2 Exclusion criteria 16
10. Study Methodology 17
10.1 Demographic and Clinical Characteristics 17
10.2 Primary Study Endpoint 17
10.2.1 Circulating Tumour Cells and Platelet Cloaking 17
10.3 Secondary Study Endpoints 17
10.3.1 Quality of Life Assessment 17
10.3.2 Systemic and localized tumour inflammation and coagulability 18
10.3.3 Expression of lethality-associated genes 18
10.3.4 Exercise Group 19
10.4 Statistical Analysis 20
10.4.1: Project 1 20

10.4.2: Project 2 ... 20
10.4.3: Project 3 ... 21
10.4.4: Project 4 ... 21
10.5: Sample Storage ... 21
11. Participant enrolment procedure ... 23
12. Study procedures ... 23
12.1 Baseline (T0) visit ... 23
12.2 Blood sampling ... 24
12.3 Telephone contact ... 25
12.4 Weekly exercise classes ... 25
12.5 Home-based daily exercise regimen ... 27
12.6 Three month (T3) clinic visit ... 28
12.7 Between T3 and T6 ... 28
12.8 Six month (T6) clinic visit ... 28
13. Confidentiality ... 30
13.1 Access and coding of data ... 30
13.2 Storage and analysis of data ... 30
Contact Details ... 31
Delivery Address ... 31
Appendices: ... 32
Appendix A: References ... 33
Appendix B: Patient Questionnaire ... 36
Appendix C: Exercise Diary ... 60
Appendix D: Borg Breathlessness Scale ... 62
Appendix E: Structured Exercise Questions ... 63
Appendix F: Amendments to Date ... 64

Abbreviations

ACSM – American College of Sports Medicine
ADT – Androgen Deprivation Therapy
BMI – Body Mass Index
CRP – C-Reactive Protein
CT – Computed Tomography
CTCs – Circulating Tumour Cells
EMT – Epithelial Mesenchymal Transition
FDA – Food and Drug Administration
HDL – High-Density Lipoprotein
HR – Heart Rate
HRR – Heart Rate Reserve
ICH GCP – International Conference on Harmonisation Good Clinical Practice
IL-6 – Interleukin 6
K2EDTA - Ethylenediaminetetraacetic AcidLDH- Lactate Dehydrogenase
MHC – Major Histocompatibility Complex
mRNA – Messenger Ribonucleic Acid
MRI – Magnetic Resonance Imaging
MS – Metabolic Syndrome
NCB – Needle Core Biopsy
NK – Natural Killer
PCR – Polymerase Chain Reaction
PDGF – Platelet-Derived Growth Factor
PI – Principal Investigator
PIN – Participant Identifier Number
PrCa – Prostate Cancer
PSA – Prostate Specific Antigen
QoL – Quality of Life
RNA – Ribonucleic Acid
RPE – Rate of Perceived Exertion
RT-PCR – Reverse Transcription Polymerase Chain Reaction
SD – Standard Deviation
T0 – Baseline
T3 – Three months
T6 – Six months
TF – Tissue Factor
TGFβ – Transforming Growth Factor Beta
TNFα – Tissue Necrosis Factor Alpha
UK – United Kingdom
VEGF – Vascular Endothelial Growth Factor

1. Abstract

1.1 Background

Obesity, known to be associated with a pro-inflammatory, pro-thrombotic humoral milieu, confers a worse prognosis in prostate cancer (PrCa). Circulating tumour cells (CTCs) are identified in the blood in advanced cancer. Their quantitation provides prognostic information. "Cloaking" of CTCs by adherent platelets impedes Natural Killer (NK)-cell clearance of CTCs from the circulation, enhancing metastatic spread. NK-cell function in blood and in solid organs is quantitatively and qualitatively reduced in obesity. Platelet cloaking may be enhanced in obesity due to the pro-inflammatory, pro-thrombotic state, and may be a mechanism for worse cancer-specific outcomes in this group. Obesity and its biochemical effects may be influenced by lifestyle changes such as exercise. Physical activity reduces levels of systemic inflammatory mediators and so aerobic exercise may represent an accessible and cost-effective means of ameliorating the pro-inflammatory effects of obesity.

1.2 Objectives

To determine whether

1. Platelet cloaking of PrCa CTCs is more prominent in men with obesity than without
2. Regular exercise can improve quality of life (QoL) and ameliorate platelet cloaking
3. The degree of platelet cloaking varies with levels of systemic and primary tumour inflammation and coagulability
4. Expression of an obesity-associated lethality gene signature leads to variation in platelet cloaking

1.3 Setting and Methods

This international multicentre prospective study will recruit 200 men with metastatic PrCa, dividing them into exposed (BMI ≥ 25) and non-exposed (BMI < 25) groups based on their body mass index (BMI). Participants will be recruited from centres in Dublin (Ireland), and London (UK), and will be randomized to exercise and control groups. Blood samples will be taken at baseline (T0) and at three (T3) and six months (T6).

Project 1: CTCs will be enumerated in the T0 samples. Adherent platelets will be quantified and compared between the exposed and non-exposed groups, and correlated with clinicopathological parameters.

Project 2: The exercise group will undertake a regular supervised aerobic exercise programme, whereas the control group will not. T3 and T6 blood samples will be assessed for CTC numbers and platelet cloaking. Changes will be compared with the T0 sample and between exposed and non-exposed and exercise and control groups. Participants will complete a detailed questionnaire to assess quality of life and other parameters at each visit.

Project 3: Blood samples will be assessed for NK-cell number and activation, markers of systemic inflammation, adipokines and serum factors related to platelet activation. The prostate needle core biopsies (NCBs) will be examined microscopically for atrophy and inflammation, by morphology and immunohistochemistry, with particular reference to NK-cells. All variables will be correlated with platelet cloaking.

Project 4: NCBs will be assessed for expression of an obesity-associated lethality gene signature (whose genes are known to play a role in obesity or platelet aggregation and coagulation), and correlated with platelet cloaking.

1.4 Impact

This study aims to elucidate a potential mechanism by which obesity confers a worse prognosis in PrCa, two increasingly prevalent diseases in the western world. We hope to show that a low-cost, accessible exercise programme can improve QoL and potentially ameliorate the effects of obesity through alterations in the systemic adipokine and inflammatory mediator profile.

2. Background and Rationale

2.1 Prostate cancer

PrCa is the second leading cause of cancer death, and many men present with locally advanced or metastatic cancer for whom curative surgery is inappropriate. For these men, increases in disease-free and overall survival and quality of life are the primary management objectives, and new therapies and assisting lifestyle alterations are increasingly needed.

2.2 Metabolic syndrome and prostate cancer

Metabolic syndrome (MS) is a constellation of risk factors for cardiovascular disease, including hypertriglyceridemia, hypertension and low high density lipoprotein (HDL)-cholesterol, with central adiposity and insulin resistance being the most important components. Between 1990-2002, Irish male obesity increased from 8% to 20%, with a further 47% overweight [1]. Male hypogonadism, due to androgen deprivation therapy (ADT) - the mainstay of treatment for locally advanced and metastatic PrCa - is an independent risk factor for the various components of metabolic syndrome [2-6]. Metabolic syndrome is present in 50% of all men undergoing long-term ADT [7], and may explain the excess non-cancer mortality in this population [8]. Obesity / MS is associated with progression but not incidence of PrCa [9], and excess body weight and high plasma C-peptide levels predispose to cancer death in men with a subsequent diagnosis of PrCa [10]. However, evidence for MS as a risk factor for the development of PrCa is not conclusive [11, 12].

Increasing evidence suggests that substantial crosstalk occurs between molecular pathways involved in inflammation, coagulation and obesity [13]. Metabolic syndrome is characterised by low-level chronic systemic inflammation. Elucidation of how these pathways interact with PrCa cells, for example through platelet cloaking will shed light on why obesity disimproves PrCa prognosis.

2.3 Circulating Tumour Cells and Prostate Cancer

Epithelial cells circulating in the blood of patients with carcinoma can be identified using various techniques including the CellSearch system, which is food and drug administration (FDA) approved for diagnostic use. Epithelial cells, defined as EpCAM+ and CD45-, rarely exceed one cell per 7.5ml of blood in patients without malignancy [14]. A drawback of CellSearch is that cells which have successfully invaded the circulation will have undergone epithelial-mesenchymal transition (EMT), often losing epithelial surface antigens such as EpCAM in the process. It is thought that

unfortunately many of the circulating cancer stem cell-like cells, which would be the fraction most likely to be biologically significant, undergo EMT as part of the metastatic cascade and thus slip beneath the radar [15]. The ScreenCell® system (*ScreenCell*, Paris, France) does not rely on EpCAM expression and so may be more useful in isolation of CTCs with EMT gene expression.

Increasing evidence suggests that numbers of CTCs may have a prognostic role in advanced PrCa. A prospective study of castration-resistant PrCa found that ≥5 CTCs per 7.5ml of blood correlated with a poor prognosis [16]. When a variety of clinical, serological and pathological parameters were considered, the model best predictive of survival was based on baseline lactate dehydrogenase (LDH), baseline CTC count and fold change in CTC count at monthly intervals [17].

2.4 Interactions between platelets and circulating tumour cells

Despite the long-recognised association between cancer and thromboembolism, it has been unclear whether the thrombocytosis often seen in patients with metastases is a consequence or cause of widespread dissemination of tumour. Accumulating evidence now shows that platelets support tumour metastasis by various mechanisms [18]. Platelets are involved in the arrest of CTCs in the vasculature and through endothelial interactions enable their extravasation. Platelets also secrete various pro-oncogenic factors including platelet-derived growth factor (PDGF) and vascular endothelial growth factor (VEGF), and mediate pro-survival signals in ovarian cancer cells [19].

Tumour cell induced platelet aggregation correlates with metastatic potential, and may be due to “cloaking” of tumour cells by adherent platelets. The interaction between platelet cloaking of CTCs and tumour cell killing by NK cells is not completely understood. Thrombocytopaenic mice exhibited reduced tumour metastatic burden when the tumour cells were NK-cell sensitive, and in-vitro studies demonstrated reduced NK tumourilytic activity when platelets aggregated around tumour cells [20]. In a later mouse model, deficiency for G α q, a G-protein critical for platelet activation, resulted in a marked decrease in experimental and spontaneous metastases [21]. This effect was eliminated in NK cell deficient mice, further supporting the hypothesis that adherent platelets may obstruct the direct cell-cell contact required for NK cell killing. Our group has recently demonstrated significant (>60%) reduction in NK cell activity (using a CD107a flow cytometry assay of NK cell activity) by platelet cloaking of cancer cells which appears to be mediated by the releasate of activated platelets (unpublished data). Another mechanism by which adherent platelets may inhibit NK cell-tumour interactions is through release of transforming growth factor beta (TGF-i) which downregulates the NK-cell activating receptor NKG2D [22]. Platelets may enable evasion of immune editing by NK-cells by conferring a “pseudonormal” phenotype on CTCs by encouraging high level surface expression of normal MHC class 1 antigen by the tumour cells [23].

Further studies show that tissue factor (TF) expression by tumour cells and the presence of serum factor XIII transglutaminase act to support metastasis by impeding NK-cell mediated clearance of tumour cells from the circulation [24, 25]. Of note, prostasomes secreted by benign and malignant prostate cells contain high concentrations of TF, with strong procoagulant effects in vitro [26]. This strongly suggests a link between TF activity and platelet cloaking of CTCs.

In a murine model, platelets activated by interaction with circulating tumour cells release adenine nucleotides which act upon the endothelial P2Y2 receptor to open the endothelial barrier and facilitate transendothelial tumour cell migration. An experimental antibody has been developed

which preferentially induces oxidative platelet fragmentation by targeting the $\beta 3$ subunit of the platelet' s fibrinogen receptor, αIIb$\beta 3$, and which reduces metastasis. Platelet-derived TGFβ and direct platelet-tumour cell contacts activate the TGFβ/Smad and NF-κB cancer cell pathways, resulting in epithelial-mesenchymal transition and enhanced metastasis in mice. All of these mechanisms require close interaction of tumour cells with platelets, interactions which can be described and quantified as platelet cloaking.

In all of these pre-clinical studies there is an association between increased platelet-tumour cell interactions and endpoints of metastasis and death in animal models, but no clinical data exist as yet relating these interactions to outcomes in human disease. The current proposed study takes the current weight of evidence that platelet interactions are important in metastasis, and attempts to make the leap to demonstrate this in a clinical population. We feel that sufficient pre-clinical suggestive evidence exists to justify the proposed translational study, and to bridge the gap between the laboratory and the clinic.

2.5 Prostate cancer and exercise

Several studies have shown that exercise may be protective against aggressive PrCa although there is no evidence that exercise protects against PrCa overall [27-30]. In PrCa patients there is solid evidence that exercise (especially group exercise) improves muscular and aerobic endurance, reduces fatigue and improves overall quality of life [31].

Physical activity reduces levels of systemic inflammatory mediators [32], such as tissue necrosis factor alpha (TNFα) and so exercise may represent an accessible and cost-effective means of ameliorating the pro-inflammatory effects of obesity. This effect of physical activity depends on type, volume and intensity and does not depend directly on weight loss [33]. We found significant changes in adipokine and inflammatory mediator concentrations in our Swedish pilot study, "Promenadgruppen". In this study men underwent a ten week walking programme involving weekly one-hour walks in small groups, and were compared to a control group. A separate published study found decreased c-reactive protein (CRP) levels following a 12-week programme of resistance and aerobic exercise [34].

2.6 Natural killer cells and obesity

NK-cell numbers in blood and in solid organs, as well as NK-cell cytotoxicity and cytokine secretion, are known to be reduced in obesity. Obese patients have lower numbers of circulating NK cells compared to non-obese patients [35]. In addition, obese people with hypertension, raised fasting glucose and unfavourable lipid profile have less NK-cells than "metabolically healthy" obese patients. Obese subjects have lower numbers of hepatic NK-cells and leptin receptor-positive cells compared with those of normal weight [36]. The NK-cell fraction of white blood cells is sensitive to exercise [37], and five-fold increases in NK concentrations following acute exercise have been noted. Brief exercise upregulates molecular pathways in circulating NK-cells associated with cancer and cell communication [38]. In healthy young men, hypoxic exercise training leads to enhanced in vitro NK-cell cytotoxicity [39].

2.7 Rationale for study

Many patients diagnosed with PrCa are not suitable for radical therapy because of the extent or grade of disease. In those patients who have potentially curable disease, obesity and its complications may make radical surgery impractical. ADT is itself a cause of obesity and the metabolic syndrome. For all of these reasons, men with PrCa who are obese are less likely to be treated with curative intent. Medical therapy is improving for the cardiovascular complications of obesity, which are the major competing cause of death in these men. As control of obesity-related cardiovascular risk factors improves, aggressiveness of PrCa becomes more important in determining the cause of mortality. We know that obese men have a worse outlook regarding cancer-related mortality than non-obese men. The combination of an aging population with an increased PrCa incidence, increasing obesity prevalence and improved management of cardiovascular risk factors means that in the future, simply put, more men are going to die as a result of the deleterious effect of overweight in advanced PrCa. Demonstration that platelet cloaking is a mechanism by which obesity disimproves PrCa survival would suggest that therapies targeted at points along the pathway of platelet activation could be efficacious. For example, adiponectin supplementation or blockade of Interleukin-6 (IL-6) or TNF α could be useful. Comparison of the expression of lethality-associated genes between the primary site and CTCs could highlight genes which are upregulated as part of the metastatic pathway, with potential for targeted therapy.

3. Study Objectives

Our overarching hypothesis is that enhanced platelet cloaking of circulating tumour cells in obese men with prostate cancer, due to increased systemic inflammation, is a mechanism underlying worse prognosis of cancer in these patients.

We aim to test the following four hypotheses, dividing the experimental and analytical work into four separate projects.

1. Platelet cloaking of circulating PrCa tumour cells is more prominent in men with obesity than without.

2. Regular exercise can ameliorate platelet cloaking.

3. The degree of platelet cloaking varies with levels of systemic and primary tumour inflammation and coagulability.

4. Expression of an obesity-associated lethality gene signature leads to variation in platelet cloaking.

For our first objective, we will recruit 200 men with metastatic PrCa, and divide them into exposed (BMI≥25) and non-exposed groups (BMI<25). Our objective will be to enumerate CTCs and quantify the degree of platelet cloaking in exposed and non-exposed groups, and to draw meaningful comparisons between the two.

For our second objective we will determine to what extent the number of CTCs and the degree of platelet cloaking varies in our exposed and non-exposed groups following a supervised exercise programme, and to compare this with a non-exercised control group.

For our third objective we will build a serological, haematological and immunological picture of the state of systemic inflammation and coagulability, and the degree of inflammation within the prostate gland. Furthermore, we intend to correlate and compare these variables with the results of our first and second objectives, in order to determine whether the number of CTCs and the degree of platelet cloaking varies with changes in the inflammatory/coagulatory milieu.

For our fourth objective we will determine whether the expression profile of a number of lethality-associated genes, known to be associated with PrCa progression, coagulation and stem-cell like phenotype, correlates with the number of CTCs and the degree of their cloaking by platelets.

CTC numbers and the degree of platelet cloaking will be common denominators which anchor these four objectives together and enable comparison between them.

Project 1: Does cloaking of CTCs vary between exposed and non-exposed groups?

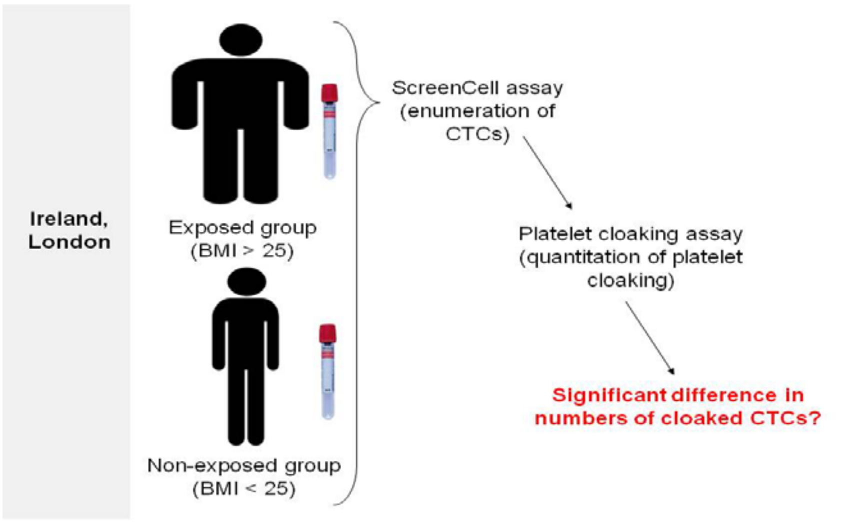

Project 2: Does six months of regular exercise reduce platelet cloaking?

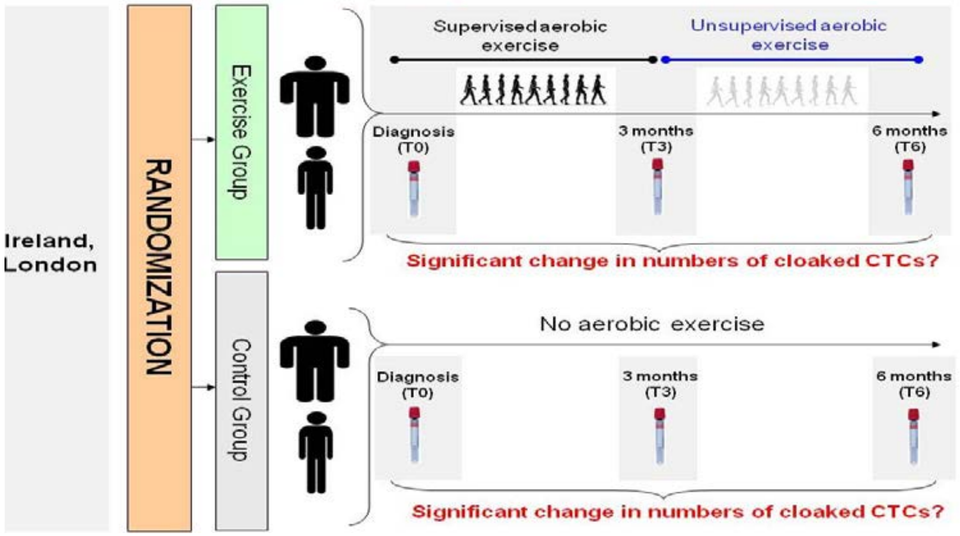

Project 3: Does the degree of platelet cloaking vary relative to markers of systemic and localised tumour inflammation and coagulability?

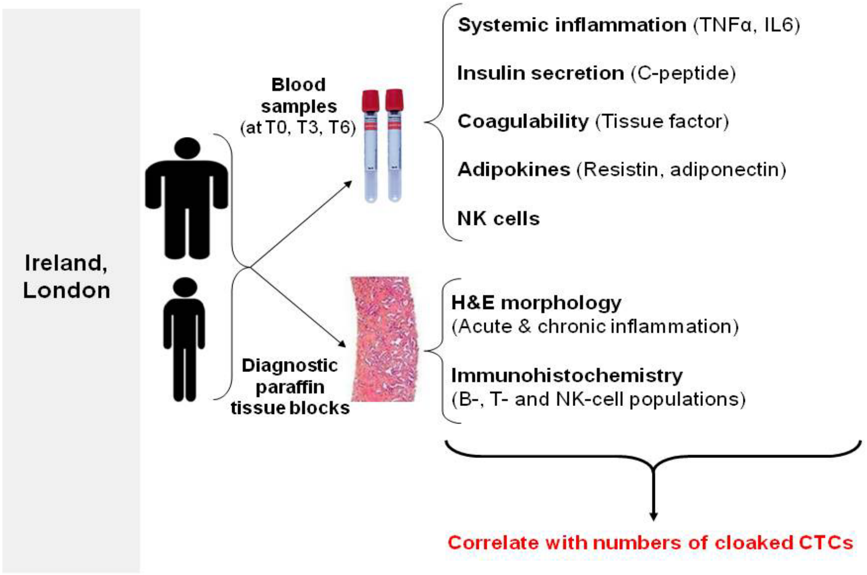

Project 4: Is overexpression of certain lethality-associated genes, known to play a role in obesity or platelet aggregation and coagulation, associated with enhanced platelet cloaking?

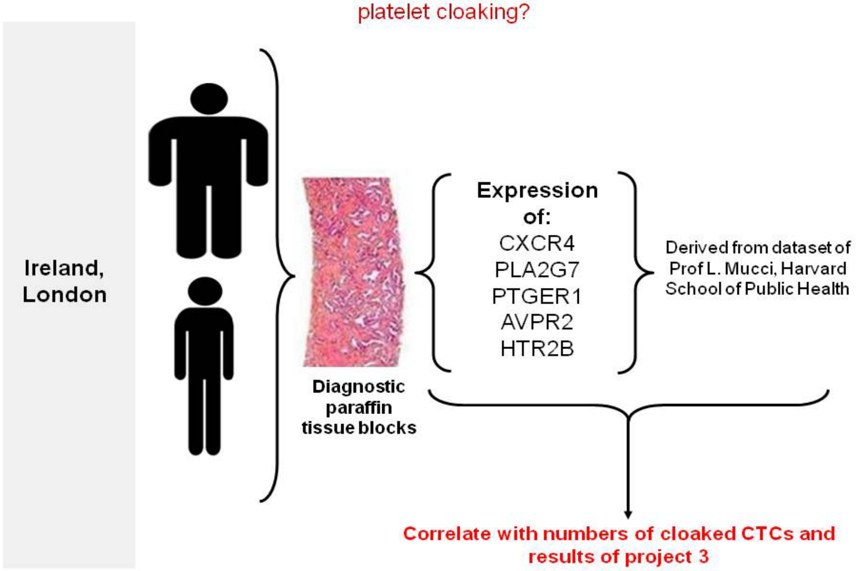

4. Study Design

This study incorporates both an observational component, with exposed and non-exposed groups defined based on BMI, and an exercise component, with randomization to exercise and control groups for a supervised exercise programme.

Participants with metastatic prostate cancer will be recruited and divided into exposed (BMI ≥ 25) and non-exposed groups (BMI < 25).

All exposed and non-exposed participants will be randomized to an exercise group or a control group, helping to minimise bias. The exercise group will participate in a six month exercise programme, comprising a weekly group exercise class and a home-based exercise programme. Participants will also be encouraged to complete activity diaries. From baseline (T0) to 3 months (T3), participants in the exercise arm will meet in small groups with a chartered physiotherapist for one hour per week. At these sessions, participants will be educated about using the Polar heart rate monitors, prescribed their target exercise intensity and complete a half hour group aerobic exercise class. From T3 to 6 months (T6) continued aerobic exercise will be encouraged but classes will not be supervised by a chartered physiotherapist.

All patients will be offered a personal exercise advice session at the study end to discuss long term compliance to physical activity guidelines. Any patients demonstrating a need for further follow up in relation to their physical activity levels will be advised to attend their GP for a referral to the GP exercise scheme.

5. Number of Participants

We will recruit 200 participants over the lifetime of the study, evenly divided between the exercise group and the control group. Recruitment, blood sampling and exercise will take place in London (Guy's Hospital) in addition to sites in Ireland. Ireland will be responsible for the recruitment of 133 patients and London (Guy's Hospital) will be responsible for recruiting 67 patients.

To calculate power of the study, we use data from our previous study of ovarian cancer cell lines which showed approximately 2% platelet adhesion [19]. A previous study of PrCa CTCs [14] showed a mean of 75 CTCs with a standard deviation of 333. If the SD of platelet adhesion in PrCa CTCs is proportionate to ovarian cancer cell lines (6.66%), then we will be able to detect a difference of 2.65% with 100 participants in both exposed and non-exposed groups, determined by independent t-testing. A standard deviation (SD) varying from 2% to 10% would enable us to detect a difference of platelet cloaking of between 0.79% and 3.9%. Research into this area is at an early stage and the clinical importance of specific incremental changes in the degree of platelet cloaking is as yet uncertain, but its elucidation is beyond the scope of this study. It should be noted that the Allard study quoted above used the CellSearch® system, which relies upon EpCAM expression and which can count only single CTCs and not microemboli or clumps. We expect to find greater numbers of CTCs using the more sensitive ScreenCell® kits.

With regard to the detection of changes in platelet cloaking with time and taking the same assumptions regarding SD of platelet adhesion in PrCa CTCs as in project 1, we will be able to detect a change of 1.8% platelet cloaking between any two time points in the 100 participants in each the exercise and the control groups, determined by paired t-testing. A SD varying from 2% to 10% would enable us to detect a difference of platelet cloaking of between 0.56% and 2.8%. Generalised linear mixed models will be employed in order to account for the correlation between multiple measurements in the same experimental subject.

6. Anticipated Enrolment Period

The study is scheduled to last for four years, initial funding was drawn down in April 2014. Enrolment commenced in November 2014 and will close at the end of March 2017. Enrolment will close twelve months before the finish date of the study (end of March 2018) in order to allow enrolled participants to complete their six months of follow up and exercise programme, and for all laboratory work and analysis to be finished before the study completion date.

7. Duration of Patient Participation

Each participant will be involved in the ExPeCT trial for six months from baseline (T0).

8. Estimated Study Completion Date

End of March 2018.

9. Participant Selection Criteria

9.1 Inclusion criteria

1. Written informed consent obtained before any study-related procedures
2. Age ≥ 18 years and male
3. Histologically confirmed diagnosis of prostate adenocarcinoma
4. M1 metastatic disease as confirmed by CT/MRI or by bone scan, excluding patients who only have nodal metastatic disease
5. Stable medical condition, including the absence of acute exacerbations of chronic illnesses, serious infections, or major surgery within 28 days prior to randomisation
6. Capable of participating safely in the proposed exercise as assessed and signed off by a treating physician involved in ExPeCT recruitment

9.2 Exclusion criteria

1. No history of radical prostatectomy
2. No previous diagnosis of any other malignant tumour (patients with non-melanoma skin cancer or carcinoma in situ of any type are not excluded provided they have undergone complete resection)

10. Study Methodology

10.1 Demographic and Clinical Characteristics

A datasheet will be completed for each participant after recruitment at baseline (T0) and at the T3 and T6 follow-up visits. Data gathered will include date of birth, anthropomorphic parameters (body weight, standing height, waist circumference), blood pressure, routine laboratory data (serum PSA, haemoglobin, white cell and platelet counts), site of metastasis and cancer-related data (stage and Gleason grade of cancer, details of current and previous systemic and radiation therapy).

10.2 Primary Study Endpoint

10.2.1 Circulating Tumour Cells and Platelet Cloaking

For each clinical review episode (at baseline and after 3 and 6 months), at least 16ml of blood will be drawn from each patient using the Vacuette® system using K_2-EDTA tubes and filtered by a ScreenCell® Cyto kit within four hours. CTC enrichment depends on vacuum-assisted filtration through a microporous membrane filter to separate CTCs from other blood cells on the basis of size. 3ml of blood is diluted in 4ml of ScreenCell® FC2 filtration buffer, incubated at room temperature for 8 minutes and passed through a ScreenCell® Cyto filtration column. Following filtration 1.6ml of 1X phosphate buffered saline is passed through the filter to wash away debris. Four-five filters will be generated for each participant, two of which will be stained with May-Grunwald Giemsa, followed by a broad-spectrum epithelial marker, and two-three reserved for platelet cloaking assays and other relevant markers. CTCs will be enumerated cytologically.

The degree of platelet adhesion to CTCs will be assessed by immunohistochemistry. Platelets will be specifically stained with antihuman CD42a antibody. The number of CTCs with adherent platelets will be counted, and the approximate number of platelets adherent to each cell will be estimated.

10.3 Secondary Study Endpoints

10.3.1 Quality of Life Assessment

All participants will complete a detailed questionnaire (Appendix B) after recruitment at baseline (T0), and again at T3 and T6. The sections of the questionnaire are as follows:

1. Background details (age at diagnosis, domiciliary situation, comorbidities, recent medications)
2. Smoking and alcohol
3. Sleep (Pittsburgh Sleep Quality Index)
4. Stress (Perceived Stress Scale – 4)
5. Depression (PHQ-9)

6. Quality of Life (FACT-P)
7. Memory and cognition
8. Physical activity
9. Diet (dairy products, meat, vitamin D)
10. Pain (Brief Pain Inventory Scale)

Some sections of the questionnaire are stand-alone validated instruments (such as FACT-P, the Functional Assessment of Cancer Therapy scales for Men with Prostate Cancer, which is designed to assess health-related quality of life in this setting). Others, such as the sections on physical activity and diet, are derived from a prostate cancer-specific questionnaire used in the large Physicians' Health Study based at Harvard University

10.3.2 Systemic and localized tumour inflammation and coagulability

This part of the project consists of measurement of systemic and prostate inflammation, markers of coagulation, cytokines and NK-cells. The substrates for this work will be blood samples taken from each participant at T0, T3 and T6, and the original diagnostic NCB paraffin tissue blocks. Flow Cytometry analysis will only take place at selected Dublin sites. Examples of the serological and haematological tests are as follows:

TNF α	Multi-plex assay
IL-6	Multi-plex assay
C-peptide	Single plex assay
Adiponectin	Single-plex assay
Resistin	Single-plex assay/Multi-plex assay
Tissue factor	Single-plex assay
NK-cells	Flow cytometry for CD45, CD56, CD3, CD69, NKB1, CD158b, CD4, CD8, CD16, CD19

The patients' diagnostic NCBs will be reviewed in order to quantify any inflammatory infiltrate present in the primary tumour. The diagnostic H&E slides will be reviewed and the infiltrate described according to a published classification system. Immunohistochemistry will be performed on the tissue block containing the most inflammation with antibodies to CD3, CD56 and CD68, in order to characterise the inflammatory infiltrate.

10.3.3 Expression of lethality-associated genes

This project will evaluate expression of selected genes known to be associated with PrCa progression, coagulation and stem-cell like phenotype in diagnostic needle core biopsies.

Sections of formalin-fixed, paraffin embedded tissue blocks will be cut from each patient's diagnostic prostate NCB specimen. These sections will be dissected by either laser capture microdissection or gross dissection.

Ribonucleic Acid (RNA) extraction from the microdissected tissue will be undertaken using the RecoverAll™ Total Nucleic Acid Isolation Kit (Life Technologies, Carlsbad CA, USA). High-ethanol washing steps ensure the recovery of smaller RNA fragments (<200 nt), including miRNA.

Gene expression profiling will be undertaken using TaqMan® Reverse Transcription Polymerase Chain Reaction (RT-PCR) (Applied Biosystems, Foster City CA, USA) in diagnostic biopsy material, using custom-designed assays designed to detect only mRNA and to traverse the exonic junction. Assays for the genes CXCR4, PLA2G7, PTGER1, AVPR2 and HTR2B will be employed. Quantitation of results of PCR will be undertaken using the $\Delta\Delta$Ct method, comparing the Ct values of the samples of interest with a control or calibrator such as a non-treated sample or RNA from normal tissue. Diagnostic material may be used for further gene expression analysis associated with obesity as part of the trial.

10.3.4 Exercise Group

The exercise group will participate in a six month moderate to vigorous intensity aerobic exercise programme, comprising a weekly class and a home-based aerobic exercise programme. Patient participation must be signed off on by a treating physician involved in recruitment to ExPeCT. Participants will also be encouraged to complete weekly activity diaries (Appendix C). From baseline (T0) to 3 months (T3), participants in the exercise arm will meet in small groups with a chartered physiotherapist for one hour per week. At these sessions, participants will be educated about using the Polar heart rate monitors, prescribed their target exercise intensity and complete a half hour group exercise class. From T3 to 6 months (T6) continued exercise will be encouraged but classes will not be supervised by a chartered physiotherapist.

Exercise intensity will be prescribed using individualised heart rate reserve (HRR) ranges in accordance with the American College of Sports Medicine (ACSM) guidelines. The following formula will be used to calculate HRR and heart rate (HR) range prescriptions: (Target % x [Maximum HR – Resting HR] + Resting HR). For each participant, age predicted maximal HR will be calculated using the following equation: (206.9 - [0.67 x age]).

Patients will also be encouraged to use the Borg Breathlessness Scale (Appendix D). Using this scale, participants will give a subjective rating of perceived exertion. It is widely used and reliable indicator to monitor and guide exercise intensity. The scale allows individuals to subjectively rate their level of exertion during exercise or exercise testing [40]. The original scale was developed in healthy individuals to correlate with exercise heart rates (e.g., RPE 15 would approximate a HR of 150 bpm), and to enable subjects to better understand terminology [41] . The Borg scale will be particularly valuable with participants on beta blockers as measures of exercise intensity are inaccurate or dampened on these medications and polar monitors may not reflect an accurate heart rate during exercise.

Exercise intensity will be progressed weekly and will be monitored using Polar heart rate monitors (Polar FT7m), with which our group has some previous experience. During months 1-3, data from the Polar heart rate monitor will be downloaded weekly to monitor adherence. The Polar FT7m heart rate monitor has the capacity to store up to 99 files and data will therefore be downloaded monthly from T3 to T6. Participants will be scheduled to attend the research centre once monthly from T3 to T6 to download data and encourage ongoing adherence to the programme. In addition, participants will receive weekly telephone contact from the ExPeCT research team from T3 to T6 to encourage adherence.

Participants will be asked to self-rate their baseline activity levels as one of three categories as per ACSM guidelines:

i) Sedentary or minimally active, not completing any moderate to vigorous activity (equivalent to poor fitness levels),

ii) Sporadic physical activity, suboptimal exercise (equivalent to fair fitness levels),

iii) Habitual physical activity, regular moderate to vigorous exercise (equivalent to average fitness levels)

Initial exercise intensity will vary depending on baseline self-rated activity levels. Exercise progression will be slightly more gradual in those who enter the programme with self-rated 'poor' fitness levels. Exercise prescription will progress in intensity and duration during months 1 and 2 of the programme to reach the target three hours per week (180 minutes/week) of moderate-to-vigorous intensity activity from month 3 onwards. This level of activity has been previously shown to be associated with a 33% reduction in all-cause mortality following prostate cancer. Participants will be encouraged to achieve this target exercise in six 30 minute sessions throughout the week. However, flexibility will be allowed to facilitate longer or shorter session to a total of 180 minutes/week. Each exercise session must be of at least 10 minutes in duration. We have previously shown that similar aerobic activity intensities can be achieved in cancer survivors through a home-based walking programme and that a Polar heart rate monitor was an acceptable means of monitoring activity intensity.

The control group will not be given specific advice regarding exercise beyond that considered usual medical care, and will not be invited to participate in the aerobic exercise group. Participants will be reviewed at three (T3) and six (T6) months following the baseline visit and anthropometric measurements and further blood samples taken. Participants assigned to the control group will be offered a personal exercise advice session following completion of the T6 assessment.

10.4 Statistical Analysis

10.4.1: Project 1

We will compare the mean and median number of cloaked platelets comparing healthy weight and overweight men using t tests and non-parametric Mann-Whitney and Wilcoxon test, respectively. We will additionally undertake linear regression models to test the association between obesity and extent of platelet cloaking, adjusting for potential confounders such as age, use of medications, smoking. In addition to comparing overweight and healthy weight men as a binary exposure, we will further model BMI as an ordinal variable (<18.5, 18.5-24.9, 25.0-27.4, 27.5-29.9, 30+) and as a continuous variable and test for linear trends with the log likelihood ratio test of nested models.

10.4.2: Project 2

We will compare measurements of platelet cloaking at baseline and months 3 and 6 follow-up time points among men randomized to the exercise and control arms, in both the exposed (BMI≥25) and non-exposed (BMI<25) groups. We will undertake intention to treat

analyses using linear mixed effect models to incorporate each biomarker for a given participant over time. In this approach, we fit a model with random intercepts representing randomly varying subject effects to account for the within-person association in biomarker levels with the appropriate link functions and covariance structure to accommodate the three timed measurements of each individual. We will additionally stratify by BMI, to look at potential effect modification.

10.4.3: Project 3

The extent of the inflammatory infiltrate in diagnostic core biopsies will be assessed as a categorical variable according to the classification system proposed by Nickel et al [42]. The amount of CD3, CD56 and CD68 staining in the infiltrate will be semi quantitatively assessed (0, 1+, 2+, 3+) as categorical variables. The serum concentration of TNF α, IL-6, C-peptide, adiponectin, resistin and tissue factor, and flow cytometry for NK cells will be quantified as continuous variables. All of the above variables will be correlated with CTC numbers and platelet cloaking using basic descriptive statistics such as Pearson correlation coefficients for continuous variables and simple t-tests for categorical variables. In the event of skewed distributions or sparse data, we will use non-parametric tests such as the Spearman correlation and Mann-Whitney U-test. Moreover, we will undertake principal component analysis to estimate the proportion of variability in platelet cloaking and CTC which is explained as a function of the obesity inflammatory biomarkers. We will model the biomarkers as principal components in the linear regression and adjust for potential confounders such as age, smoking, and other factors.

10.4.4: Project 4

Gene expression profiling will be undertaken using TaqMan® RT-PCR (Applied Biosystems, Foster City CA, USA) in diagnostic biopsy material, using custom-designed assays designed to detect only mRNA and to traverse the exonic junction. Assays for the genes CXCR4, PLA2G7, PTGER1, AVPR2 and HTR2B will be employed. Quantitation of results of PCR will be undertaken using the $\Delta\Delta$Ct method, comparing the Ct values of the samples of interest with a control or calibrator such as a non-treated sample or RNA from normal tissue.

We will use generalized linear regression models to examine whether obesity is associated with expression of each of the five markers in tumour tissue, adjusting for potential confounders such as age, smoking status, as well as clinical features. We will dichotomize obesity as BMI greater or less than 25, and also model BMI as a continuous variable and examine tests for trend. We will assess expression of each marker with respect to extent of platelet cloaking (High, intermediate and low). We will further create a gene score by ranking individuals across expression of each gene in tertiles, assigning points for each marker as (lowest tertile = 0; middle tertile = 1; upper tertile=2) and calculating a summary score.

10.5: Sample Storage

Serum and plasma samples will be stored in a designated temperature monitored freezer at -80°C in Room 1.22 in the Central Pathology Lab, St James's Hospital. Screencell filters will be stored short-term at 4°C and then moved to -20°C for long term storage. The 4°C fridge

and -20°C freezer are both located in Room 1.18 in the Central Pathology Lab, St James's Hospital. Biological samples will be securely stored for up to 10 years with the option of requesting ethical permission for a prolonged storage time. Samples from London will be shipped on dry ice (serum and plasma) and using cool packs at 4°C (Screencell filters) to Room 1.01 Sir Patrick Dun Lab, Central Pathology Lab, St James's Hospital, Dublin.

11. Participant enrolment procedure

Potential patients will be screened and enrolled on the study on the basis of the inclusion / exclusion criteria specified in the protocol. Screening of potential patients will be undertaken by staff at the medical oncology clinics at each recruiting site as well as members of the ExPeCT research team who have been delegated this task by the PI. Any queries about eligibility will be addressed directly to the Chief Investigator.

Staff from oncology clinics or a member of the ExPeCT research team delegated the task by the PI will approach potential patients and must give each patient an information sheet. Informed consent will be obtained by clinic staff or a member of the ExPeCT research team according to the requirements of International Conference on Harmonisation (ICH)- Good Clinical Practice (GCP).

Upon registration of new participants, a signature confirming eligibility for the trial must be obtained from a treating physician involved in ExPeCT recruitment. Participants recruited from St James's Hospital and Tallaght Hospital will be registered at their respective sites. Participants recruited from Beaumont, Mater Misericordiae and St Luke's will be referred to St James's Hospital and will be allocated a hospital medical record number. Registration of these patients and further participation in the trial will be conducted within the Clinical Research Facility in St James's Hospital.

Each registered patient will receive a unique participant identifier number (PIN) which is obtained from the ExPeCT research team by contacting 087-6577927. This PIN is separate from their medical record number.

Potential participants may also be identified by the ExPeCT research team retrospectively from previous clinic lists. These patients may be contacted with trial information through the post by a member of the ExPeCT research team and the Chief Investigator. Patients are invited to contact a member of the ExPeCT research team if interested in participating in the trial and will then be given a clinic appointment and proceed through the enrolment procedure as outlined above.

In order to ensure random allocation of participants to each study group, the computer programme Graphpad will be used to randomly assign PINs to the control or exercise groups. Only members of the ExPeCT research team will have access to this computer programme. If a participant chooses to withdraw from the study, all data obtained up to the point of withdrawal will be carried forward unless requested otherwise.

12. Study procedures

12.1 Baseline (T0) visit

Following recruitment of a new participant, the recruiting healthcare professional or member of the ExPeCT research team who has been delegated this task by the PI will complete a study datasheet with the assistance of the participant using their newly-assigned PIN. This will include measurement of height, weight and waist circumference. The participant will be provided with a questionnaire,

which can be completed either during his clinic visit with or without the assistance of the recruiting healthcare professional/member of the ExPeCT research team, or taken home to be completed later and posted to the ExPeCT research team. Patients may also be asked to complete a structured interview session with the chartered physiotherapist exploring attitudes towards exercise (Appendix E).

12.2 Blood sampling

Blood (16-39ml) will be drawn by the recruiting healthcare professional or a qualified member of the ExPeCT research team delegated this task by the PI. If the recruiting healthcare professional is performing the blood draw, contact should be made with a member of the ExPeCT research team before a blood sample is taken to determine whether a member of the ExPeCT research team is available to collect and filter the sample. The ExPeCT research team can arrange with the patient to take the blood sample subsequently

The protocol for obtaining blood samples is as follows:

1. Blood samples must be filtered in the research lab **WITHIN 4 HOURS** of being drawn from the patient in order to provide useful results. This will be performed in the research laboratory in St James's Hospital (Ireland Sites) and Guys hospital (London site) by a member of the ExPeCT research team.

2. If possible, to avoid unnecessary additional venepuncture, participants should have their research blood samples drawn as part of the same blood draw as any clinical samples which are to be obtained.

3. In order to reduce possible contamination of the research samples by skin cells, the research blood samples should be the last blood tubes filled during the blood draw, **AFTER** any clinical samples which are to be obtained.

4. If no clinical blood samples are to be taken, please discard at least the first 1ml of blood which is drawn before filling the research blood tubes.

5. The following tubes should be drawn as per the table below:

Order of Priority	Sample Type	Tube Type	Minimum Volume required
1	Serum	Z Serum Clot Activator	5ml
2	Whole Blood/CTCs	K2EDTA	16-30*ml
3	Plasma	9NC Coagulation Sodium Citrate 3/2%	3.5ml

*16ml at all sites except St James's Hospital where 24-30ml is required

6. After filling with blood **EACH K2-EDTA TUBE AND THE CITRATE TUBE SHOULD BE INVERTED AT LEAST 10 TIMES**. This mixes the anticoagulant with the blood and ensures that it will not clot. If this step is omitted the blood samples are likely to be useless. .

7. Label each tube with the Participant Identified Number and the date and **TIME** of blood draw. All relevant details pertaining to the blood draw should also be recorded in the biological sample log.

8. K2EDTA blood samples are kept at room temperature and filtered as soon as possible. The serum tube should be allowed to stand for 1hour prior to processing. The citrate tube can be processed immediately.

12.3 Telephone contact

Within one week of the T0 clinic visit, the ExPeCT research team will contact the newly recruited participant by telephone in order to discuss the following:

1. Thank the participant for agreeing to take part and give him an opportunity to withdraw if he wishes
2. If the participant has been randomised to the exercise group, arrange for the participant to attend his first exercise class, receive his heartrate monitor and plan out his exercise regimen.
3. If the T0 blood sample was not obtained at the recruitment visit, arrange a suitable time for same
4. Participants who have been randomised into the intervention group will be informed that they will be provided with parking vouchers, if applicable to their site, which will cover the time of each weekly exercise session.

During the T3-T6 period, a weekly telephone call or email from the ExPeCT research team will emphasise the importance of maintaining adherence to the exercise plan although participants will no longer be attending weekly exercise classes.

12.4 Weekly exercise classes

Participants recruited in St James's Hospital, St. Luke's, Mater Misericordiae and Beaumont will undertake exercise in the Clinical Research Facility in St James's Hospital. Participants recruited in Tallaght Hospital and Guy's Hospital will undertake exercise in collaboration with the physiotherapy departments at their local sites. During the first class the participants will receive an introduction to the format of the exercise and will be educated on safe exercise practices and strategies to monitor exercise exertion.

Exercise prescription will be individually prescribed based on self-rated baseline activity levels.
Participants will be asked to rate their fitness levels based on baseline habitual activity patterns as per the following categories:

i) Sedentary or minimally active, not completing any moderate to vigorous activity (equivalent to poor fitness levels),

ii) Sporadic physical activity, suboptimal exercise (equivalent to fair fitness levels),

iii) Habitual physical activity, regular moderate to vigorous exercise (equivalent to average fitness levels)

Participants with self-rated 'poor' fitness levels (category i) will commence the programme at an aerobic intensity of 40-50% HRR. Those with self-rated 'fair' fitness levels (category ii) will commence the programme at an aerobic intensity of 50-60% HRR and those with self-rated 'average' fitness levels (category iii) will commence the programme 55-65% HRR. Participants will be fitted with individual Polar heart rate monitor to monitor aerobic exercise intensity and will be educated on the use of the monitor. Each participant will receive one monitor to keep for their personal use during the programme.

The volume of exercise completed during the programme will increase weekly from T0 to T3 through increases in both the duration of exercise completed and the intensity of exercise prescribed as per Table 1. Exercise progression will be managed by the lead chartered physiotherapist supervising the exercise class.

**Supervised Exercise Classes**		**Exercise Intensity (%HRR) Baseline Fitness Groups**			**Duration**
		**Poor**	**Fair**	**Average**	**Minutes**
**Month 1**	Week 1	40-50%	50-60%	55-65%	20
	Week 2	40-50%	50-60%	55-65%	20
	Week 3	45-55%	55-65%	60-70%	20
	Week 4	45-55%	55-65%	60-70%	30
**Month 2**	Week 5	50-60%	60-70%	65-75%	30
	Week 6	50-60%	60-70%	65-75%	30
	Week 7	55-65%	65-75%	65-75%	30
	Week 8	55-65%	65-75%	65-75%	30
**Month 3**	Week 9	60-70%	65-75%	65-75%	30
	Week 10	60-70%	65-75%	65-75%	30
	Week 11	60-75%	65-75%	65-75%	30
	Week 12	60-75%	65-75%	65-75%	30

The group will meet once weekly in a designated gym within a hospital setting. Exercise intensity will be achieved through completion of aerobic exercise such as treadmill walking. In order to minimize the risk of physical injury to participants, only those regarded as medically fit to undertake to exercise programme will be invited to participate. Forms of aerobic exercise undertaken at the supervised exercise classes will specifically avoid activities which may be associated with higher risk (e.g. the use of rowing machines in participants with lumbar spinal metastases). Walking on treadmills is a low-risk exercise activity.

During T0 to T3 participants will meet weekly to complete the exercise programme. Participants will also complete the home-based exercise programme during this time as described in section 12.5 below.

12.5 Home-based daily exercise regimen

In addition to the supervised weekly aerobic exercise class described in section 12.4 above, participants will be instructed to complete a home based aerobic exercise programme. The aerobic intensity of the home based exercise programme will correspond weekly to that prescribed in the exercise class. Participants will wear the Polar heart rate monitors during all home exercise to provide them with feedback as to their target exercise zone. Data from the monitors will be downloaded by the chartered physiotherapist at the start of each exercise class to monitor adherence to the prescribed protocol. Participants will also be encouraged to complete daily exercise diaries. The number of exercise sessions completed per week will progress throughout the programme to the desired 6 sessions of 30 minutes of moderate intensity aerobic activity. While participants will be encouraged to complete six sessions of 30 minutes duration weekly, flexibility will be allowed to facilitate less frequent longer exercise sessions.

The duration and frequency of the home exercise programme sessions is outlined in Table 2 below.

**Home based walking programme**		**Exercise Intensity (%HRR) Baseline Fitness Groups**			**Time**	
		**Poor**	**Fair**	**Average**	**Days/week**	**Duration (minutes)**
**Month 1**	Week 1	40-50%	50-60%	55-65%	2	20
	Week 2	40-50%	50-60%	55-65%	3	20
	Week 3	45-55%	55-65%	60-70%	3	20
	Week 4	45-55%	55-65%	60-70%	3	30
**Month 2**	Week 5	50-60%	60-70%	65-75%	3	30
	Week 6	50-60%	60-70%	65-75%	4	30
	Week 7	55-65%	65-75%	65-75%	4	30
	Week 8	55-65%	65-75%	65-75%	5	30
**Month 3**	Week 9	60-70%	65-75%	65-75%	5	30
	Week 10	60-70%	65-75%	65-75%	5	30
	Week 11	60-75%	65-75%	65-75%	5	30
	Week 12	60-75%	65-75%	65-75%	5	30
**Month 4**	Weeks 13-16	60-75%	65-75%	65-75%	6	30
**Month 5**	Weeks 17-20	60-75%	65-75%	65-75%	6	30
**Month 6**	Weeks 12-24	60-75%	65-75%	65-75%	6	30

12.6 Three month (T3) clinic visit

Participants will be invited to attend outpatients three months after baseline (T0) and the trial datasheet and questionnaire will again be completed. Blood samples will be obtained in the same fashion as for the T0 visit.

12.7 Between T3 and T6

During this period participants in the exercise arm will no longer attend weekly classes, but nonetheless they will be encouraged to continue their daily exercise regimen with their heartrate monitors. Participants will attend the location of the exercise classes once per month in order for the ExPeCT research team to download data from the heart rate monitors.

12.8 Six month (T6) clinic visit

Participants will be invited to attend outpatients six months after baseline (T0) and the trial datasheet and questionnaire will again be completed. Blood samples will be obtained in the same fashion as for the T0 visit. All patients will be offered a personal exercise advice session at the study end to discuss long term compliance to physical activity guidelines. Any patients demonstrating a need for further follow up in relation to their physical activity levels will be advised to attend their GP for a referral to the GP exercise scheme.

After this visit participants will be thanked for their involvement and discharged from the study.

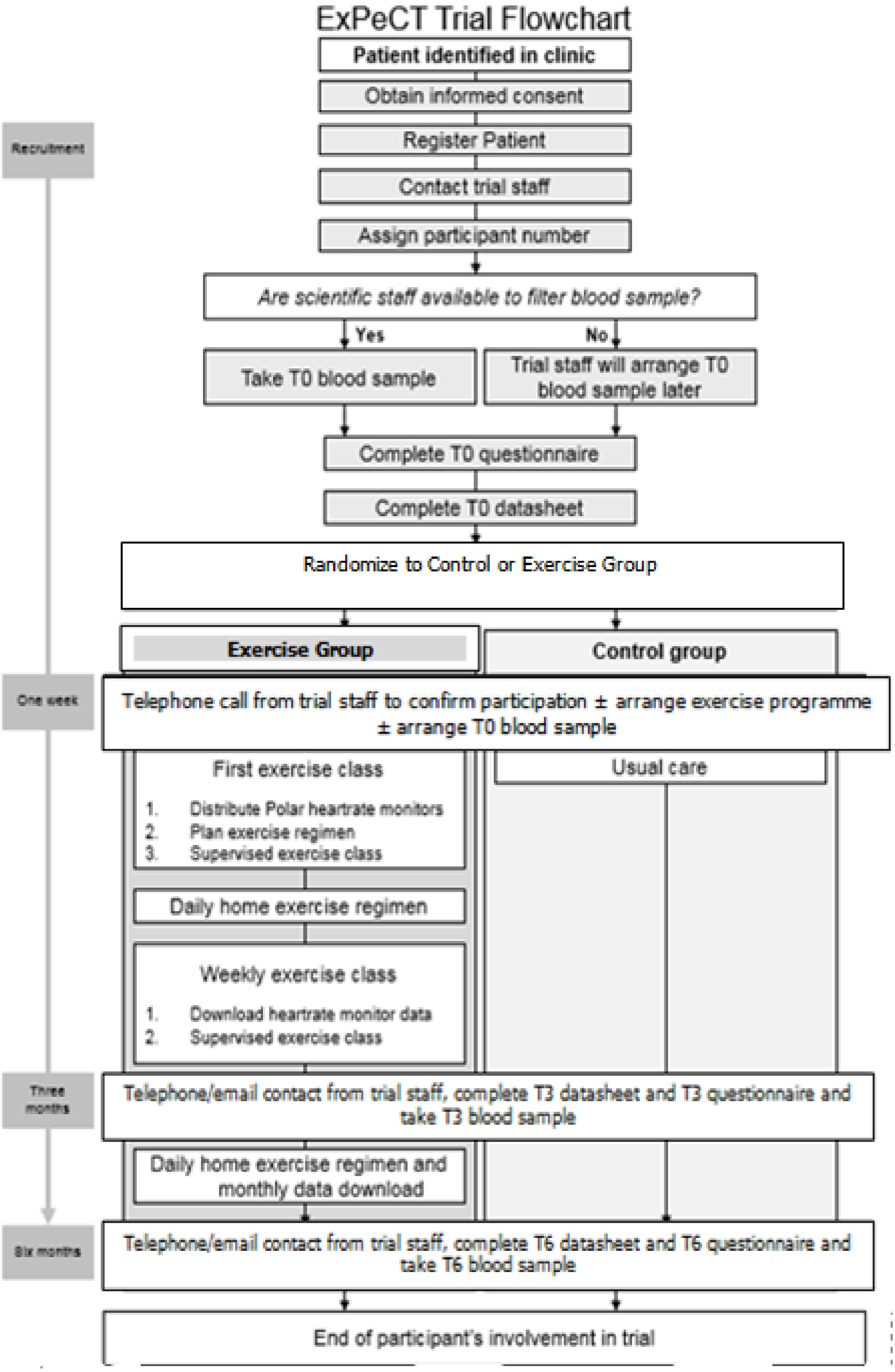
ExPeCT Trial Flowchart
Patient identified in clinic
Obtain informed consent
Register Patient
Contact trial staff
Assign participant number
Are scientific staff available to filter blood sample?
Yes
No
Take T0 blood sample
Trial staff will arrange T0 blood sample later
Complete T0 questionnaire
Complete T0 datasheet
Randomize to Control or Exercise Group
Exercise Group
Control group
Recruitment
One week
Telephone call from trial staff to confirm participation ± arrange exercise programme ± arrange T0 blood sample
First exercise class
1. Distribute Polar heartrate monitors
2. Plan exercise regimen
3. Supervised exercise class
Usual care
Daily home exercise regimen
Weekly exercise class
1. Download heartrate monitor data
2. Supervised exercise class
Three months
Telephone/email contact from trial staff, complete T3 datasheet and T3 questionnaire and take T3 blood sample
Daily home exercise regimen and monthly data download
Six months
Telephone/email contact from trial staff, complete T6 datasheet and T6 questionnaire and take T6 blood sample
End of participant's involvement in trial

13. Confidentiality

13.1 Access and coding of data

The ExPeCT research team led by Prof. Stephen Finn will be the only people with access to the data collected in the course of this project, which will be in electronic and paper-based hard copy formats. All data will be coded by use of a unique Participant Identifier Number and the ExPeCT research team based in St. James's Hospital will retain the key to re-identify data.

13.2 Storage and analysis of data

Paper questionnaires, data record forms and consent forms will be stored in a locked filing cabinet at St James's Hospital, in a locked office in a security-card-protected building with restricted access. Participants' details and results of experiments will be stored on a secure password-protected computer in St. James's Hospital and protected Trinity and National University of Ireland Galway servers. Multiple copies of the data will not be made.
Data analysis will be performed in St. James's Hospital by the in-house bioinformatics team and other members of the ExPeCT research team.

At the end of the study period, when all analysis is complete, data will be retained by the ExPeCT research team. Data will be securely stored for up to 10 years with the option of requesting ethical permission for a prolonged storage time.

Contact Details

Name	Email	Role
Dr Brian Hayes	Brian.hayes@hse.ie	Consultant Histopathologist
Dr Emer Guinan	emguinan@tcd.ie	Research Fellow
Dr Orla Casey	Orla.casey@tcd.ie	Research Fellow
Grainne Sheill	sheillg@tcd.ie	PhD Student/Chartered Physiotherapist
Lauren Brady	Bradyl4@tcd.ie	PhD Student
Dr Tatjana Vlajnic	Tatjana.vlajnic@usb.ch	Pathologist
Carrie Burgess	Carrie.burgess@kcl.ac.uk	Research Nurse
Fee Cahill	Fidelma.cahill@kcl.ac.uk	Research Nurse

Delivery Address
Trinity College Dublin
Dept. of Histopathology
Room 1.01 Sir Patrick Dun Lab,
Central Pathology Lab,
St James's Hospital,
Dublin 8.

Appendices:

Appendix A: References
Appendix B: Patient Questionnaire
Appendix C: Exercise Diary
Appendix D: Borg Breathlessness Scale
Appendix E: Structured Exercise Interview
Appendix F: Amendments to Date

Appendix A: References

1. McCarthy, S.N., M.J. Gibney, and A. Flynn, *Overweight, obesity and physical activity levels in Irish adults: evidence from the North/South Ireland food consumption survey.* Proc Nutr Soc, 2002. **61**(1): p. 3-7.
2. Smith, J.C., et al., *The effects of induced hypogonadism on arterial stiffness, body composition, and metabolic parameters in males with prostate cancer.* J Clin Endocrinol Metab, 2001. **86**(9): p. 4261-7.
3. Dockery, F., et al., *Testosterone suppression in men with prostate cancer leads to an increase in arterial stiffness and hyperinsulinaemia.* Clin Sci (Lond), 2003. **104**(2): p. 195-201.
4. Smith, M.R., H. Lee, and D.M. Nathan, *Insulin sensitivity during combined androgen blockade for prostate cancer.* J Clin Endocrinol Metab, 2006. **91**(4): p. 1305-8.
5. Keating, N.L., A.J. O'Malley, and M.R. Smith, *Diabetes and cardiovascular disease during androgen deprivation therapy for prostate cancer.* J Clin Oncol, 2006. **24**(27): p. 4448-56.
6. Keating, N.L., et al., *Diabetes and cardiovascular disease during androgen deprivation therapy: observational study of veterans with prostate cancer.* J Natl Cancer Inst, 2010. **102**(1): p. 39-46.
7. Braga-Basaria, M., et al., *Metabolic syndrome in men with prostate cancer undergoing long-term androgen-deprivation therapy.* J Clin Oncol, 2006. **24**(24): p. 3979-83.
8. Van Hemelrijck, M., et al., *Absolute and relative risk of cardiovascular disease in men with prostate cancer: results from the Population-Based PCBaSe Sweden.* J Clin Oncol, 2010. **28**(21): p. 3448-56.
9. Smith, M.R., et al., *Diabetes and mortality in men with locally advanced prostate cancer: RTOG 92-02.* J Clin Oncol, 2008. **26**(26): p. 4333-9.
10. Ma, J., et al., *Prediagnostic body-mass index, plasma C-peptide concentration, and prostate cancer-specific mortality in men with prostate cancer: a long-term survival analysis.* Lancet Oncol, 2008. **9**(11): p. 1039-47.
11. Laukkanen, J.A., et al., *Metabolic syndrome and the risk of prostate cancer in Finnish men: a population-based study.* Cancer Epidemiol Biomarkers Prev, 2004. **13**(10): p. 1646-50.
12. Tande, A.J., E.A. Platz, and A.R. Folsom, *The metabolic syndrome is associated with reduced risk of prostate cancer.* Am J Epidemiol, 2006. **164**(11): p. 1094-102.
13. Zhang, N. and D.A. Lawrence, *Tissue factor and obesity, a two-way street.* Nat Med, 2011. **17**(11): p. 1343-4.
14. Allard, W.J., et al., *Tumor cells circulate in the peripheral blood of all major carcinomas but not in healthy subjects or patients with nonmalignant diseases.* Clin Cancer Res, 2004. **10**(20): p. 6897-904.
15. Chen, C.L., et al., *Single-cell analysis of circulating tumor cells identifies cumulative expression patterns of EMT-related genes in metastatic prostate cancer.* Prostate, 2013. **73**(8): p. 813-26.
16. de Bono, J.S., et al., *Circulating tumor cells predict survival benefit from treatment in metastatic castration-resistant prostate cancer.* Clin Cancer Res, 2008. **14**(19): p. 6302-9.
17. Scher, H.I., et al., *Circulating tumour cells as prognostic markers in progressive, castration-resistant prostate cancer: a reanalysis of IMMC38 trial data.* Lancet Oncol, 2009. **10**(3): p. 233-9.
18. Gay, L.J. and B. Felding-Habermann, *Contribution of platelets to tumour metastasis.* Nat Rev Cancer, 2011. **11**(2): p. 123-34.

19. Egan, K., et al., *Platelet adhesion and degranulation induce pro-survival and pro-angiogenic signalling in ovarian cancer cells.* PLoS One, 2011. **6**(10): p. e26125.
20. Nieswandt, B., et al., *Lysis of tumor cells by natural killer cells in mice is impeded by platelets.* Cancer Res, 1999. **59**(6): p. 1295-300.
21. Palumbo, J.S., et al., *Platelets and fibrin(ogen) increase metastatic potential by impeding natural killer cell-mediated elimination of tumor cells.* Blood, 2005. **105**(1): p. 178-85.
22. Kopp, H.G., T. Placke, and H.R. Salih, *Platelet-derived transforming growth factor-beta down-regulates NKG2D thereby inhibiting natural killer cell antitumor reactivity.* Cancer Res, 2009. **69**(19): p. 7775-83.
23. Placke, T., et al., *Platelet-derived MHC class I confers a pseudonormal phenotype to cancer cells that subverts the antitumor reactivity of natural killer immune cells.* Cancer Res, 2012. **72**(2): p. 440-8.
24. Palumbo, J.S., et al., *Tumor cell-associated tissue factor and circulating hemostatic factors cooperate to increase metastatic potential through natural killer cell-dependent and-independent mechanisms.* Blood, 2007. **110**(1): p. 133-41.
25. Palumbo, J.S., et al., *Factor XIII transglutaminase supports hematogenous tumor cell metastasis through a mechanism dependent on natural killer cell function.* J Thromb Haemost, 2008. **6**(5): p. 812-9.
26. Babiker, A.A., et al., *Prothrombotic effect of prostasomes of metastatic cell and seminal origin.* Prostate, 2007. **67**(4): p. 378-88.
27. Giovannucci, E.L., et al., *A prospective study of physical activity and incident and fatal prostate cancer.* Arch Intern Med, 2005. **165**(9): p. 1005-10.
28. Patel, A.V., et al., *Recreational physical activity and risk of prostate cancer in a large cohort of U.S. men.* Cancer Epidemiol Biomarkers Prev, 2005. **14**(1): p. 275-9.
29. Nilsen, T.I., P.R. Romundstad, and L.J. Vatten, *Recreational physical activity and risk of prostate cancer: A prospective population-based study in Norway (the HUNT study).* Int J Cancer, 2006. **119**(12): p. 2943-7.
30. Johnsen, N.F., et al., *Physical activity and risk of prostate cancer in the European Prospective Investigation into Cancer and Nutrition (EPIC) cohort.* Int J Cancer, 2009. **125**(4): p. 902-8.
31. Keogh, J.W. and R.D. MacLeod, *Body composition, physical fitness, functional performance, quality of life, and fatigue benefits of exercise for prostate cancer patients: a systematic review.* J Pain Symptom Manage, 2012. **43**(1): p. 96-110.
32. Ho, S.S., et al., *Effects of chronic exercise training on inflammatory markers in Australian overweight and obese individuals in a randomized controlled trial.* Inflammation, 2013. **36**(3): p. 625-32.
33. Balducci, S., et al., *Anti-inflammatory effect of exercise training in subjects with type 2 diabetes and the metabolic syndrome is dependent on exercise modalities and independent of weight loss.* Nutr Metab Cardiovasc Dis, 2010. **20**(8): p. 608-17.
34. Galvao, D.A., et al., *Combined resistance and aerobic exercise program reverses muscle loss in men undergoing androgen suppression therapy for prostate cancer without bone metastases: a randomized controlled trial.* J Clin Oncol, 2010. **28**(2): p. 340-7.
35. Lynch, L.A., et al., *Are natural killer cells protecting the metabolically healthy obese patient?* Obesity (Silver Spring), 2009. **17**(3): p. 601-5.
36. Lautenbach, A., et al., *Human obesity reduces the number of hepatic leptin receptor (ob-R) expressing NK cells.* Endocr Res, 2011. **36**(4): p. 158-66.
37. Timmons, B.W. and T. Cieslak, *Human natural killer cell subsets and acute exercise: a brief review.* Exerc Immunol Rev, 2008. **14**: p. 8-23.

38. Radom-Aizik, S., et al., *Impact of brief exercise on peripheral blood NK cell gene and microRNA expression in young adults.* J Appl Physiol (1985), 2013. **114**(5): p. 628-36.
39. Wang, J.S. and T.P. Weng, *Hypoxic exercise training promotes antitumour cytotoxicity of natural killer cells in young men.* Clin Sci (Lond), 2011. **121**(8): p. 343-53.
40. Borg, G.A., *Borg's Perceived Exertion and Pain Scales*. 1998, Champaign, IL: Human Kinetics.
41. Borg, G.A., *Psychophysical bases of perceived exertion.* Med Sci Sports Exerc, 1982. **14**(5): p. 377-81.
42. Nickel, J.C., et al., *Consensus development of a histopathological classification system for chronic prostatic inflammation.* BJU Int, 2001. **87**(9): p. 797-805.

Appendix B: Patient Questionnaire

ExPeCT Trial:

Exercise, Prostate Cancer and Circulating Tumour Cells

Patient Questionnaire

Participant number: _______________

Date of Birth: _______________

Date of Questionnaire: _______________

Is this the first, second or third time you have filled in this questionnaire?

1st ☐ 2nd ☐ 3rd ☐

Section 1 – Background details

1. What age were you when you were diagnosed with prostate cancer? _________

2. How would you describe your race / ethnic background?

   White/Caucasian ☐ Black/Afro-Caribbean ☐ Asian ☐

3. What is your current marital status?

   Married ☐ Divorced/Separated ☐ Widowed ☐

4. What is your current living arrangement?

   Alone ☐ With wife ☐ With other family ☐

   Assisted Living ☐ [illegible]ursing ☐ Other ☐

5. What is your current work status?

   Full-time ☐ Part-time ☐ Retired ☐

   Disabled ☐ Unemployed ☐

For the following questions please circle the appropriate answer:

6. Have you been diagnosed with any of the following medical conditions?'

(a) High blood pressure	YES	NO
(b) Diabetes mellitus	YES	NO
(c) High cholesterol	YES	NO
(d) Myocardial infarction (heart attack)	YES	NO
(e) Angina pectoris	YES	NO
(f) Atrial fibrillation	YES	NO
(g) Congestive heart failure	YES	NO

7. Have you regularly taken any of these medications *in the last two years*?

(a) Non-steroidal anti-inflammatory drugs (NSAIDs)

(i) Aspirin	YES	NO
(ii) Ibuprofen (e.g. Advil, Motrin, Nuprin, Medipren)	YES	NO
(iii) Other: ___________________	YES	NO

(b) "Statin" cholesterol-lowering drugs

(i) Lovastatin (e.g. Mevacor, Altocor)	YES	NO
(ii) Simvastatin (e.g. Zocor)	YES	NO
(iii) Pravastatin (e.g. Pravachol, Pravigard)	YES	NO
(iv) Atorvastatin (e.g. Lipitor)	YES	NO

(c) Beta blocker drugs

(i) Metoprolol (e.g. Lopressor, Toprol)	YES	NO
(ii) Atenolol (e.g. Tenormin)	YES	NO

(iii) Nadolol (e.g. Corgard)	YES	NO
(iv) Other: ___________________________________	YES	NO

(d) Antidepressants: Selective serotonin reuptake inhbitors (SSRIs)

(i) Citalopram (e.g. Celexa)	YES	NO
(ii) Escitalopram (e.g. Lexapro)	YES	NO
(iii) Fluoxetine (e.g. Prozac)	YES	NO
(iv) Paroxetine (e.g. Paxil)	YES	NO
(v) Sertraline (e.g. Zoloft)	YES	NO
(vi) Fluvoxamine (e.g. Luvox)	YES	NO

(e) Other antidepressants

(i) Amitriptyline (e.g. Elavil, Endep)	YES	NO
(ii) Imipramine(e.g. Tofranil)	YES	NO
(iii) Nortriptyline (e.g. Pamelor)	YES	NO
(iv) Other: ____________________	YES	NO

(f) Sleeping tablets

(i) Diazepam (e.g. Valium)	YES	NO
(ii) Alprazolam (e.g. Xanax)	YES	NO
(iii) Lorazepam(e.g. Ativan)	YES	NO
(iv) Chlordiazepoxide (e.g. Librium)	YES	NO

(g) Diabetes medications

(i) Insulin	YES	NO
(ii) Metformin	YES	NO
(iii) Rosiglitazone (e.g. Avandia)	YES	NO
(iv) Pioglitazone (e.g. Actos)	YES	NO

Section 2 – Smoking and alcohol

Please circle the most appropriate answer:

1. Do you currently smoke cigarettes? (exclude pipe or cigars) YES NO

2. If you answered YES to question 2.1, how many cigarettes do you smoke per day?

   1-4 *5-14* *15-24* *25-34* *35-44* *45 or more*

3. In a typical week over the past three months, on how many days did you consume an alcoholic beverage of any type?

   No days *1 day per week* *2 days per week* *3 days per week*

   4 days per week *5 days per week* *6 days per week* *7 days per week*

4. In a typical month, what is the largest number of drinks of beer, wine and / or spirits you have in one day?

   None *1-2 drinks per day* *3-5 drinks per day*

   6-9 drinks per day *10-14 drinks per day* *15 or more drinks per day*

5. In a typical week during the past three months, how often did you drink alone?

   Never / I don't drink *Less than once a month*

   Once or twice per week *Three to five times per week*

   Almost every day

6. If you answered question 2.5 with anything other than “never”, on those days when you drank alone, how many drinks did you typically consume?

   1 Drink *2 Drinks* *3-4 Drinks*

   5-6 Drinks *More than 7 drinks*

Section 3 – Sleep (Pittsburgh Sleep Quality Index)

INSTRUCTIONS:
The following questions relate to your usual sleep habits during the past month only. Your answers should indicate the most accurate reply for the majority of days and nights in the past month. Please answer all questions.

1. During the past month, what time have you usually gone to bed at night?

   BED TIME ___________

2. During the past month, how long (in minutes) has it usually taken you to fall asleep each night?

   NUMBER OF MINUTES ___________

3. During the past month, what time have you usually gotten up in the morning?

   GETTING UP TIME ___________

4. During the past month, how many hours of actual sleep did you get at night? (This may be different than the number of hours you spent in bed.)

   HOURS OF SLEEP PER NIGHT ___________

For each of the remaining questions, check the one best response. Please answer all questions.

5. During the past month, how often have you had trouble sleeping because you . . .

a) Cannot get to sleep within 30 minutes

Not during the past month_____	Less than once a week_____	Once or twice a week_____	Three or more times a week_____

b) Wake up in the middle of the night or early morning

Not during the past month_____	Less than once a week_____	Once or twice a week_____	Three or more times a week_____

c) Have to get up to use the bathroom

Not during the past month_____	Less than once a week_____	Once or twice a week_____	Three or more times a week_____

d) Cannot breathe comfortably

Not during the past month_____ Less than once a week_____ Once or twice a week_____ Three or more times a week_____

e) Cough or snore loudly

Not during the past month_____ Less than once a week_____ Once or twice a week_____ Three or more times a week_____

f) Feel too cold

Not during the past month_____ Less than once a week_____ Once or twice a week_____ Three or more times a week_____

g) Feel too hot

Not during the past month_____ Less than once a week_____ Once or twice a week_____ Three or more times a week_____

h) Had bad dreams

Not during the past month_____ Less than once a week_____ Once or twice a week_____ Three or more times a week_____

i) Have pain

Not during the past month_____ Less than once a week_____ Once or twice a week_____ Three or more times a week_____

j) Other reason(s), please describe____

____

How often during the past month have you had trouble sleeping because of this?

Not during the past month_____ Less than once a week_____ Once or twice a week_____ Three or more times a week_____

6. During the past month, how would you rate your sleep quality overall?

Very good ___________

Fairly good ___________

Fairly bad ___________

Very bad ___________

7. During the past month, how often have you taken medicine to help you sleep (prescribed or "over the counter")?

Not during the past month_____	Less than once a week_____	Once or twice a week_____	Three or more times a week_____

8. During the past month, how often have you had trouble staying awake while driving, eating meals, or engaging in social activity?

Not during the past month_____	Less than once a week_____	Once or twice a week_____	Three or more times a week_____

9. During the past month, how much of a problem has it been for you to keep up enough enthusiasm to get things done?

No problem at all	___________
Only a very slight problem	___________
Somewhat of a problem	___________
A very big problem	___________

10. Do you have a bed partner or room mate?

No bed partner or room mate	___________
Partner/room mate in other room	___________
Partner in same room, but not same bed	___________
Partner in same bed	___________

If you have a room mate or bed partner, ask him/her how often in the past month you have had . . .

a) Loud snoring

Not during the past month_____	Less than once a week_____	Once or twice a week_____	Three or more times a week_____

b) Long pauses between breaths while asleep

Not during the past month_____	Less than once a week_____	Once or twice a week_____	Three or more times a week_____

c) Legs twitching or jerking while you sleep

Not during the past month_____	Less than once a week_____	Once or twice a week_____	Three or more times a week_____

d) Episodes of disorientation or confusion during sleep

Not during the past month_____ Less than once a week_____ Once or twice a week_____ Three or more times a week_____

e) Other restlessness while you sleep; please describe_______________________________________

____

Not during the past month_____ Less than once a week_____ Once or twice a week_____ Three or more times a week_____

Section 4 – Stress (Perceived Stress Scale – 4)

The questions in this section ask you about your feelings and thoughts *during the last month*.

In each case, please indicate your response by placing an "X" over the circle representing *how often* you felt or thought a certain way.

	Never	Almost never	Sometimes	Fairly often	Very often
1. In the last month, how often have you felt that you were unable to control the important things in your life?	O	O	O	O	O
2. In the last month, how often have you felt confident about your ability to handle your personal problems?	O	O	O	O	O
3. In the last month, how often have you felt that things were going your way?	O	O	O	O	O
4. In the last month, how often have you felt difficulties were piling up so high that you could not overcome them?	O	O	O	O	O

Section 5 – Depression (PHQ-9)

Over the last *2 weeks*, how often have you been bothered by any of the following problems? *(use "✓" to indicate your answer)*	Not at all	Several days	More than half the days	Nearly every day
**1.** Little interest or pleasure in doing things	0	1	2	3
**2.** Feeling down, depressed, or hopeless	0	1	2	3
**3.** Trouble falling or staying asleep, or sleeping too much	0	1	2	3
**4.** Feeling tired or having little energy	0	1	2	3
**5.** Poor appetite or overeating	0	1	2	3
**6.** Feeling bad about yourself—or that you are a failure or have let yourself or your family down	0	1	2	3
**7.** Trouble concentrating on things, such as reading the newspaper or watching television	0	1	2	3
**8.** Moving or speaking so slowly that other people could have noticed. Or the opposite — being so figety or restless that you have been moving around a lot more than usual	0	1	2	3
**9.** Thoughts that you would be better off dead, or of hurting yourself	0	1	2	3
add columns		+	+	
(Healthcare professional: For interpretation of TOTAL, please refer to accompanying scoring card). TOTAL:				

**10.** If you checked off *any problems*, how *difficult* have these problems made it for you to do your work, take care of things at home, or get along with other people?	
	Not difficult at all ______
	Somewhat difficult ______
	Very difficult ______
	Extremely difficult ______

Section 6 – Quality of Life (FACT-P)

Below is a list of statements that other people with your illness have said are important. **Please circle or mark one number per line to indicate your response as it applies to the past 7 days.**

	PHYSICAL WELL-BEING	Not at all	A little bit	Some-what	Quite a bit	Very much
GP1	I have a lack of energy	0	1	2	3	4
GP2	I have nausea	0	1	2	3	4
GP3	Because of my physical condition, I have trouble meeting the needs of my family	0	1	2	3	4
GP4	I have pain	0	1	2	3	4
GP5	I am bothered by side effects of treatment	0	1	2	3	4
GP6	I feel ill	0	1	2	3	4
GP7	I am forced to spend time in bed	0	1	2	3	4

	SOCIAL/FAMILY WELL-BEING	Not at all	A little bit	Some-what	Quite a bit	Very much
GS1	I feel close to my friends	0	1	2	3	4
GS2	I get emotional support from my family	0	1	2	3	4
GS3	I get support from my friends	0	1	2	3	4
GS4	My family has accepted my illness	0	1	2	3	4
GS5	I am satisfied with family communication about my illness	0	1	2	3	4
GS6	I feel close to my partner (or the person who is my main support)	0	1	2	3	4
Q1	*Regardless of your current level of sexual activity, please answer the following question. If you prefer not to answer it, please mark this box* ☐ *and go to the next section.*					
GS7	I am satisfied with my sex life	0	1	2	3	4

Please circle or mark one number per line to indicate your response as it applies to the past 7 days.

	**EMOTIONAL WELL-BEING**	**Not at all**	**A little bit**	**Some-what**	**Quite a bit**	**Very much**
GE1	I feel sad	0	1	2	3	4
GE2	I am satisfied with how I am coping with my illness	0	1	2	3	4
GE3	I am losing hope in the fight against my illness	0	1	2	3	4
GE4	I feel nervous	0	1	2	3	4
GE5	I worry about dying	0	1	2	3	4
GE6	I worry that my condition will get worse	0	1	2	3	4

	**FUNCTIONAL WELL-BEING**	**Not at all**	**A little bit**	**Some-what**	**Quite a bit**	**Very much**
GF1	I am able to work (include work at home)	0	1	2	3	4
GF2	My work (include work at home) is fulfilling	0	1	2	3	4
GF3	I am able to enjoy life	0	1	2	3	4
GF4	I have accepted my illness	0	1	2	3	4
GF5	I am sleeping well	0	1	2	3	4
GF6	I am enjoying the things I usually do for fun	0	1	2	3	4
GF7	I am content with the quality of my life right now	0	1	2	3	4

Please circle or mark one number per line to indicate your response as it applies to the past 7 days.

	ADDITIONAL CONCERNS	Not at all	A little bit	Some-what	Quite a bit	Very much
C2	I am losing weight	0	1	2	3	4
C6	I have a good appetite	0	1	2	3	4
P1	I have aches and pains that bother me	0	1	2	3	4
P2	I have certain parts of my body where I experience pain	0	1	2	3	4
P3	My pain keeps me from doing things I want to do	0	1	2	3	4
P4	I am satisfied with my present comfort level	0	1	2	3	4
P5	I am able to feel like a man	0	1	2	3	4
P6	I have trouble moving my bowels	0	1	2	3	4
P7	I have difficulty urinating	0	1	2	3	4
BL2	I urinate more frequently than usual	0	1	2	3	4
P8	My problems with urinating limit my activities	0	1	2	3	4
BL5	I am able to have and maintain an erection	0	1	2	3	4

Section 7 – Memory and Cognition

Please circle the most appropriate answer.

Over the past three months:

1. Do you have more trouble than usual remembering recent events?	YES	NO
2. Do you have more trouble than usual remembering a short list of items, such as a shopping list?	YES	NO
3. Do you have trouble remembering things from one second to the next?	YES	NO
4. Do you have difficulty in understanding or following spoken instructions?	YES	NO
5. Do you have more trouble than usual following a group conversation or a plot in a TV programme due to your memory?	YES	NO
6. Do you have trouble finding your way around familiar streets?	YES	NO

Section 8 – Physical activity
Please circle the most appropriate answer:

1. Do you have difficulty climbing a flight of stairs or walking eight blocks (about a mile) due to physical impairment?

   YES NO

2. What is your usual walking pace outdoors?
   Please tick:

Unable to walk eight blocks or climb a flight of stairs due to physical impairment.	
Easy, Casual *(Less than 2mph)*	
Normal, average *(2-2.9mph)*	
Brisk pace *(3-3.9mph)*	
Very brisk/striding *(4mph or faster)*	

3. How many flights of stairs (not steps) do you climb daily? (Do not include time spent on exercise machines)

   No flights 1-2 flights 3-4 flights

   5-9 flights 10-14 flights 15 or more flights

4. In an average week, on how many days do you usually exercise (include brisk walking or more strenuous activity)?

   None One Two Three

   Four Five Six Seven

5. During the last three months, what was your average total time per week at each of these activities?

	NONE	1-4 minutes	5-19 minutes	20-39 minutes	40-80 minutes	1.5 hours	2-3 hours	4-6 hours	7-10 hours	11-20 hours	21-30 hours	31-40 hours	40+ hours
**Walking to work or for exercise** *(including golf)*													
**Jogging** *(slower than 10 minutes per mile)*													
**Running** *(10 minutes per mile or faster*)													
**Bicycling** *(including stationary machine)*													
**Lap swimming**													
**Tennis**													
**Squash or racquetball**													
**Other aerobic exercise** *(e.g. exercise classes etc)*													

**Other lower intensity exercise** *(e.g. yoga, bowling)*													
**Moderate outdoor work** *(e.g. gardening, yardwork)*													
	NONE	1-4 minutes	5-19 minutes	20-39 minutes	40-80 minutes	1.5 hours	2-3 hours	4-6 hours	7-10 hours	11-20 hours	21-30 hours	31-40 hours	40+ hours
**Heavy outdoor work** *(e.g. digging, chopping)*													
Weight training / resistance exercises for **arms**													
Weight training / resistance exercises for **legs**													
**Standing or walking around work**													
**Standing or walking around home**													

**Sitting at work or commuting**													
**Sitting at home while watching TV / DVD**													
**Other sitting at home**													

Section 9 – Diet

1. What type of milk do you use most often?

None Skimmed Milk Low-fat Milk

Whole milk/ Full fat milk Super / Fortified Milk Soya Milk

Other (Please specify ____)

2. How much milk do you drink each day?

None Half Pint (284ml) One Pint (568ml)

One Litre More than one litre

3. Please indicate how often, on average, over the past three months, you have eaten or drank the specified amount of each of the following foods and drinks.

		None	< 1 a month	1-3 a month	1 a week	2-4 a week	5-6 a week	1 a day	2-3 a day	4-5 a day	6+ a day
1.	Cup of tea with caffeine *(8 oz.) – includes green tea*										
2.	Yoghurt *(1 cup)*										
3.	Cottage or ricotta cheese *(1/2 cup)*										
4.	Cream cheese *(1 ounce)*										
5.	Other regular cheese, alone or as part of a dish *(1 slice or 1 ounce)*										
6.	Other low-fat cheese, alone or as part of a dish *(1 slice or 1 ounce)*										
7.	Ice cream *(1/2 cup)*										
8.	Processed meats, sausage, salami, bologna, hotdog *(1*										

	slice or piece)										
9.	Beef, pork, lamb: as a sandwich or mixed dish*, e.g. stew, casserole, lasagna*										
10.	Beef, pork, lamb: as a main dish*, e.g. steak, roast*										

4. **(a)** Have you regularly taken vitamin D since your prostate cancer diagnosis?

YES NO

(b) If you answered "yes" to question 9.4(a), please indicate your daily dose, the year you started taking vitamin D, the duration for which you have taken vitamin D since your diagnosis, and whether you are currently taking vitamin D.

(i) Daily dose: ___________________IU

(ii) Year you started taking vitamin D: __ __ __ __

(iii) Duration of taking vitamin D:

(Only include time spent taking vitamin D after you were diagnosed with prostate cancer):

Less than 6 months 6-12 months 1-2 Years 3+ Years

(iv) Are you currently taking vitamin D?

YES NO

Section 10 – Brief Pain Inventory

Brief Pain Inventory (Short Form)

1. Throughout our lives, most of us have had pain from time to time (such as minor headaches, sprains, and toothaches). Have you had pain other than these everyday kinds of pain today?

☐ Yes ☐ No

2. On the diagram, shade in the areas where you feel pain. Put an X on the area that hurts the most.

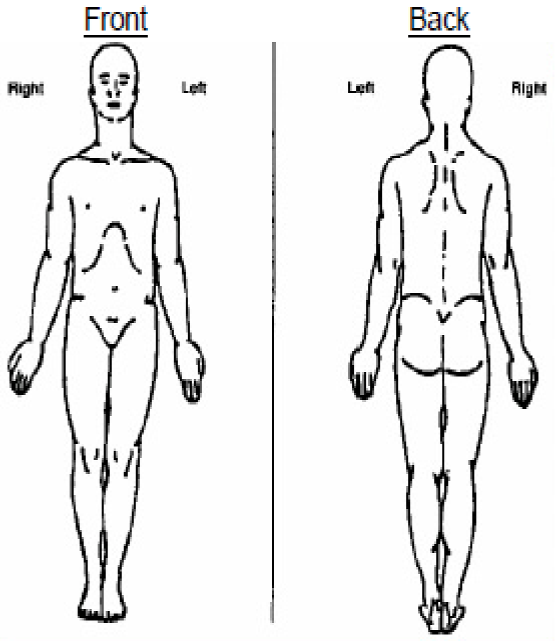

3. Please rate your pain by marking the box beside the number that best describes your pain at its worst in the last 24 hours.

☐ 0 ☐ 1 ☐ 2 ☐ 3 ☐ 4 ☐ 5 ☐ 6 ☐ 7 ☐ 8 ☐ 9 ☐ 10

No Pain — Pain As Bad As You Can Imagine

4. Please rate your pain by marking the box beside the number that best describes your pain at its least in the last 24 hours.

☐ 0 ☐ 1 ☐ 2 ☐ 3 ☐ 4 ☐ 5 ☐ 6 ☐ 7 ☐ 8 ☐ 9 ☐ 10

No Pain — Pain As Bad As You Can Imagine

5. Please rate your pain by marking the box beside the number that best describes your pain on the average.

☐ 0 ☐ 1 ☐ 2 ☐ 3 ☐ 4 ☐ 5 ☐ 6 ☐ 7 ☐ 8 ☐ 9 ☐ 10

No Pain — Pain As Bad As You Can Imagine

6. Please rate your pain by marking the box beside the number that tells how much pain you have right now.

☐ 0 ☐ 1 ☐ 2 ☐ 3 ☐ 4 ☐ 5 ☐ 6 ☐ 7 ☐ 8 ☐ 9 ☐ 10

No Pain — Pain As Bad As You Can Imagine

7. What treatments or medications are you receiving for your pain?

8. In the last 24 hours, how much relief have pain treatments or medications provided? Please mark the box below the percentage that most shows how much relief you have received.

0%	10%	20%	30%	40%	50%	60%	70%	80%	90%	100%
☐ No Relief	☐	☐	☐	☐	☐	☐	☐	☐	☐	☐ Complete Relief

9. Mark the box beside the number that describes how, during the past 24 hours, pain has interfered with your:

A. General Activity

☐ 0 Does Not Interfere ☐ 1 ☐ 2 ☐ 3 ☐ 4 ☐ 5 ☐ 6 ☐ 7 ☐ 8 ☐ 9 ☐ 10 Completely Interferes

B. Mood

☐ 0 Does Not Interfere ☐ 1 ☐ 2 ☐ 3 ☐ 4 ☐ 5 ☐ 6 ☐ 7 ☐ 8 ☐ 9 ☐ 10 Completely Interferes

C. Walking ability

☐ 0 Does Not Interfere ☐ 1 ☐ 2 ☐ 3 ☐ 4 ☐ 5 ☐ 6 ☐ 7 ☐ 8 ☐ 9 ☐ 10 Completely Interferes

D. Normal Work (includes both work outside the home and housework)

☐ 0 Does Not Interfere ☐ 1 ☐ 2 ☐ 3 ☐ 4 ☐ 5 ☐ 6 ☐ 7 ☐ 8 ☐ 9 ☐ 10 Completely Interferes

E. Relations with other people

☐ 0 Does Not Interfere ☐ 1 ☐ 2 ☐ 3 ☐ 4 ☐ 5 ☐ 6 ☐ 7 ☐ 8 ☐ 9 ☐ 10 Completely Interferes

F. Sleep

☐ 0 Does Not Interfere ☐ 1 ☐ 2 ☐ 3 ☐ 4 ☐ 5 ☐ 6 ☐ 7 ☐ 8 ☐ 9 ☐ 10 Completely Interferes

G. Enjoyment of life

☐ 0 Does Not Interfere ☐ 1 ☐ 2 ☐ 3 ☐ 4 ☐ 5 ☐ 6 ☐ 7 ☐ 8 ☐ 9 ☐ 10 Completely Interferes

Appendix C: Exercise Diary

Exercise Diary

Week ___

Your target heart rate for the next week is between

_____ and _____ beats per minute.

Date:	Duration:	Type of Exercise:	Max Heart Rate:

Please complete _____ additional sessions(s) this week of _____mins duration, as prescribed in your class

Exercise Information:

Warm-up:

Your warm-up should be similar to that preformed in the exercise class. Your warm-up should be ____ mins in duration. You should exercise at < 11 on the RPE scale as explained in class.

Aerobic Component:

The aerobic component of the programme is again set at the same intensity as performed in the exercise class this week. You should exercise for___ mins in duration at a target heart rate of between ___ and ___beats per minute. You should exercise at 12-13 on the RPE scale.

You do not need to reach the higher heart rate target and you should always exercise at a comfortable level.

Cool-down:

Again your cool-down should be similar to the cool down performed in the exercise class. Your cool-down should last ___ mins in duration. Again your cool down should correspond to <11 on the RPE scale.

Following cool down complete your lower leg stretches as on your exercise handout.

Please be advised to cease any exercise or stretching if pain is produced.

Appendix D: Borg Breathlessness Scale

Rating of Perceived Exertion (RPE) Category Scale

6
7 Very, very light
8
9 Very light
10
11 Fairly light
12
13 Somewhat hard
14
15 Hard
16
17 Very hard
18
19 Very, very hard
20

Borg G. Borg's Perceived Exertion and Pan Scales. *Champaign, IL: Human Kinetics, 1998.*

Patient Instructions for Borg Scale:
"This is a scale that asks you to rate the difficulty of your breathing. It starts at number 0 where your breathing is causing you no difficulty at all and progresses through to number 10 where your breathing difficulty is maximal. How much difficulty is your breathing causing you right now?"

Appendix E: Structured Exercise Questions

Structured Exercise Interview Questions:

1. What has been your relationship with exercise to date?
2. Do you feel that you get enough exercise as you need?
3. What factors, if any, do you think prevent you from exercising?
4. Is there any particular activity that you've stopped doing/think you shouldn't do since your diagnosis?
5. Do you generally do more, less or the same amount of sport and recreational physical activity as you did this time last year?
6. How do your family feel about you participating in exercise?
7. Do you feel you are less/more active than other people your age?

Do you see your exercise/activity levels changing in the future? Why?

Appendix F: Amendments to Date

Amendment 1.1 18th November 2014

Administrative changes such as grammar, typos etc.

Section 11: Participant Enrolment Procedure – Previous clinic lists can be screened and possible participants contacted retrospectively by post

Section 12.1: Baseline (T0) visit – Inclusion of exercise interview and changes to recruitment process

Amendment 1.3 27th August 2015

Administrative changes such as grammar, typos etc.

Addition of names to ExPeCT team

Addition of new table of contents

Insertion of abbreviation list

Addition of contact list and appendices

Section 1.3: Settings and Methods - Removal of Orebro as a study site

Section 3: Study Objectives - Diagrams changed to aerobic exercise instead of walking

Section 4: Study Design - Change of research co-ordinator to chartered physiotherapist

Section 5: Number of Participants – Addition of multiple Irish sites, removal of Swedish site

Section 9: Inclusion Criteria – Modified to specify form of metastatic disease and include signature of a treating physician

Section 10.1: Demographic and Clinical Characteristics – Inclusion of site of metastasis to data collected

Section 10.2: Subjective Assessment – Pain section included in detailed questionnaire

Section 10.3: Circulating Tumour Cells and Platelet Cloaking – Adjustments made to filtering and staining protocols

Section 10.4: Systemic and localized tumour inflammation and coagulability – Specific assays removed

Section 10.5: Expression of lethality-associated genes – Removal of LCM detailed process, replaced with option of gross dissection

Section 10.6: Exercise Group – Changes in exercise structure, type and inclusion of Borg Scale

Section 10.7.5: Sample Storage – Addition of section pertaining to the storage of biological samples

Section 11: Participant Enrolment Procedure – Changes to screening and recruiting of prospective patients and overhaul of randomisation procedure

Section 12.1: Baseline (T0) visit – Inclusion of exercise interview and changes to recruitment process

Section 12.2 Blood Sampling – Alteration in blood volume to match the different sites and changes to blood collection protocol

Section 12.3: Telephone Contact – Inclusion of parking vouchers, Inclusion of telephone contact at T3-T6

Section 12.4: Weekly Exercise Classes – Addition of new sites and exercise centres and explanation of forms of exercise to be undertaken

Section 12.5: Home-Based Daily Exercise Regimen – Changed from walking to aerobic exercise

Section 12.7: Between T3 and T6 - Movement of telephone contact to section 12.3

Section 13.2: Storage and Analysis of Data – Period of data storage and retention changed

Amendment 1.4 14TH September 2015

Addition of a personal exercise advice session at the study end and referral to the GP exercise scheme if patient demonstrates a need for further follow up.

Section 4 & 12.8: Study Design & 6 Month (T6) Clinic Visit –

> "All patients will be offered a personal exercise advice session at the study end to discuss long term compliance to physical activity guidelines. Any patients demonstrating a need for further follow up in relation to their physical activity levels will be advised to attend their GP for a referral to the GP exercise scheme."
